# Equity in the Distribution of Regulatory PM_2.5_ Monitors

**DOI:** 10.1021/acs.est.4c12915

**Published:** 2025-07-23

**Authors:** Zoé Haskell-Craig, Kevin P. Josey, Patrick L. Kinney, Priyanka deSouza

**Affiliations:** † Department of Biostatistics, 550517 New York University School of Global Public Health, New York, New York 10003, United States; ‡ Department of Biostatistics & Informatics, 144805 Colorado School of Public Health, Aurora, Colorado 80045, United States; § 1846 Department of Environmental Health, Boston University School of Public Health, Boston, Massachusetts 02118, United States; ∥ Urban and Regional Planning Department, University of Colorado Denver, Denver, Colorado 80202, United States

**Keywords:** EPA monitors;, PM_2.5_;, environmental justice;, monitor network design;, air pollution

## Abstract

Unequal exposure to air pollution by race and socioeconomic status is well-documented in the U.S. However, relatively little research has examined inequities in the collection of PM_2.5_ data, creating a critical gap in understanding which neighborhood exposures are represented in these data sets. In this study, we use multilevel models with random intercepts by county and state, stratified by urbanicity, to assess associations between six key environmental justice (EJ) attributes (%AIAN, %Asian, %Black, %Hispanic, %White, %Poverty) at the census tract level and proximity to the nearest regulatory monitor. Our results show that most EJ attributes exhibit weak or statistically insignificant associations with monitor proximity, with the exception of %Poverty, where higher poverty levels are significantly linked to closer monitors in urban (β = −0.88, 95% CI = [−0.93, −0.83]) and farther monitor distances in rural (β = 0.6, 95%CI = [0.49, 0.71]) areas. While the U.S. EPA’s siting criteria may be effective in ensuring equitable monitor distribution in many contexts, the low density of monitors in rural areas may impact the accuracy of national-level air pollution monitoring.

## Graphical abstract

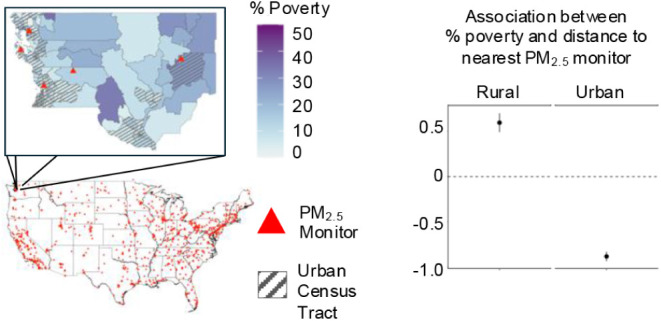

## 1 Introduction

Disparities in PM_2.5_ exposure by race/ethnicity and socioeconomic status (SES) are well-documented^1–5^ and have persisted for over 30 years, despite overall declines in air pollution concentrations.^6,7^ There is extensive literature on the disproportionate PM_2.5_ exposure in under-resourced, low-income communities of color compared to wealthier, Whiter neighborhoods. Compounded by increased social stressors and higher baseline disease rates, these inequities exacerbate the health impacts of pollution in these neighborhoods.^8^ However, less work has focused on inequities in air pollution data collection and monitoring. To our knowledge, only four studies have examined the key environmental justice (EJ) concern of whether the U.S. Environmental Protection Agency’s (EPA) network of PM_2.5_ monitors is equitably distributed.^9–12^ Monitor placement determines from which communities we have air pollution data (e.g., under-representation of certain neighborhoods in the EPA PM_2.5_ concentration data sets) and the distribution of benefits conferred by the presence of regulatory monitors (e.g., enforcement of regulatory standards).^13^ For instance, counties with monitors that record high pollution levels are subject to restrictions and experienced, on average, a decrease in PM_2.5_ between 0.47 and 2.77 μg/m^3^ over a three-year period.^14^ This study examines the association between EJ attributes at the census tract level­(race/ethnicity, SES) and the location of the nearest regulatory PM_2.5_ monitoring station across the EPA’s network.

Regulatory air quality monitoring is conducted through two networks: the State or Local Air Monitoring Stations (SLAMS) network (approximately 1449 active monitors) and the Interagency Monitoring of Protected Visual Environments (IMPROVE) network (159 PM_2.5_ monitors). SLAMS are managed by local government entities (e.g., Florida Department of Environmental Protection, Linn County Health Department, Twenty-Nine Palms Band of Mission Indians), with local regulators responsible for determining monitor locations. However, federal guidelines govern the number of monitors per county (by population size and recent NAAQS attainment status) and outline the categories of sites that should be represented in the network (e.g., locations capturing the highest concentration of PM_2.5_ in the area or the general background pollution levels).^15^ Federal requirements also stipulate that regions with extra SLAMS monitors must place at least one monitor in a community at high risk for poor air quality (e.g., near a port).^15^ In addition, each state must also maintain at least one regional background and one regional transport site (typically located in sparsely populated areas).^15^ The IMPROVE network surveys federally protected lands (e.g., national parks), with monitors sited away from local pollution sources.^16^ Despite the different siting criteria, both networks are charged with the goals to (1) provide timely information regarding air pollution, (2) support compliance with air quality standards, and (3) facilitate research into the effects of air pollution.^17^

However, the three goals of the regulatory monitoring network each suggest a different siting strategy. For instance, regulating air pollution to meet NAAQS levels requires placing monitors in areas with high PM_2.5_ concentrations, whereas health research may require a network of monitors that represent the typical air pollution exposure of the U.S. population.^18^ In addition, there may be too few monitors to accurately capture the variability in pollution that often exists at the census tract or block-group area level.^12,19,20^ As such, the data recorded from these monitors may be unrepresentative of the pollution levels experienced by many individuals. There is also some evidence that industry emitters game the system. For instance, one study demonstrated that air quality is worse on days during which regulatory monitors are not collecting data.^21^ Furthermore, research has shown that regulatory monitor data may be particularly unrepresentative of the exposures faced by individuals of color and those living in poverty.^12^ A case study conducted on all EPA air quality system (AQS) monitors in Tampa, Florida, found that individuals who are Black, Hispanic, and those living in poverty disproportionately lived closer to sources of air pollution and further from monitoring sites than the overall county population. The measurement bias induced by regulatory monitor siting may be the result of local agencies trying to decrease the discovery of pollution “hotspots” by strategically siting monitors away from sources of pollution, which are over-represented in low-income communities and communities of color.^22,23^ Grainger and Schreiber (2019) find that counties slated to receive a new NO_2_ monitor are more likely to site monitors away from pollution hotspots, except for hotspots in neighborhoods that are Whiter and wealthier.^10^ Hotspot avoidance may ultimately contribute to documented disparities in misclassifications of NAAQS attainment across racial and ethnic groups.^14^

Unequal monitoring may perpetuate disparities in exposure misclassification from models developed from this data set. EPA regulatory monitors comprise the largest network of high-quality ground-based PM_2.5_ data that are routinely used for a variety of purposes, including to calibrate chemical transport models,^24^ as the ground-based measurements for developing PM_2.5_ measurements from remote sensing-derived aerosol optical depth (AOD),^25,26^ to assess the accuracy^27^ and calibration of low-cost sensors,^28^ and as the input data set for empirical (“land-use regression”) models—including for the six major models producing national estimates.^29^ As Bechle et al. (2023)^29^ state, “Whatever strengths or weaknesses exist in using EPA monitors (and their locations) for empirical models, those likely impact all of the [empirical] models.” Measurements from the network and exposure assessments from these models are regularly used in health research assessing the impact of PM_2.5_ on cardiovascular disease,^30^ low birth weight,^31^ and diabetes, among others.^32–37^

Despite the critical role of regulatory PM_2.5_ monitors in pollutant data collection and monitoring, little work has investigated whether there are systematic racial/ethnic or income disparities in the location of these monitors. Previous work has focused on whether such monitors robustly capture pollution variability^38^ or hotspots.^39^ To our knowledge, only three studies have assessed the network at a national level from an environmental justice perspective.^10,11,40^ Grainger and Schreiber (2019)^10^ focused on the placement of new NO_2_ monitors, rather than the existing monitor network as a whole. Miranda et al. (2011)^40^ assessed the presence/absence of PM_2.5_ and O_3_ monitors at a county level, without considering their location with respect to subcounty variability in socioeconomic and demographic characteristics. Pedde and Adar (2024)^11^ addressed concerns with the use of regulatory data as input in empirical models by investigating the representativeness of monitor locations with respect to covariates typically included as predictors in these models (e.g., population density, land use, etc.). In particular, they stratify by race and calculate the fraction of the U.S. population that lives outside (above/below the maximum/minimum value) of the numeric range of covariate values. Other work on disparities in monitor placement has focused on low-cost sensors, such as PurpleAir monitors, rather than on the more costly monitors that the EPA deploys.^41–43^ Our goal is to investigate whether there are disparities by race/ethnicity and SES in the census tract distance to the nearest PM_2.5_ monitor across the SLAMS and IMPROVE networks.

## 2 Methods

### 2.1 Data

Regulatory PM_2.5_ monitor locations were obtained from the U.S. EPA’s Outdoor Air Quality data set of annual pollutant concentrations for 2019, 2022, and 2023.^44^ We conduct the main analysis using data from 2022, representing fairly typical exposure patterns (Figure S5). In a sensitivity analysis, we replicate the models for 2019 (prepandemic) and 2023 (the most recent year with complete data). For this analysis, we included only monitors recording local PM_2.5_ conditions, resulting in a final sample size of 969 monitors. For each of the 84,285 census tracts in the contiguous U.S. (excluding Alaska and Hawaii), we computed the distance from the 2020 population-weighted centroid, obtained from the IPUMS National Historical Geographic Information System,^45^ to (a) the nearest monitor site and (b) the nearest monitor site located within the same county. For the latter, we excluded tracts located in counties without a monitoring site. We use the distance to the nearest monitor as a proxy for monitor coverage for the following reasons. Simply considering the tract within which a monitor is located (“unit-hazard coincidence” approach) ignores neighboring tracts when monitors are located near the boundary.^46^ Assigning monitors to tracts if they fall within a specific radius (“buffer” approach) requires specifying a buffer size, which may not reflect differing drivers of pollution and pollutant transport conditions across the country.

Considering axes of EJ widely used in the literature,^47–49^ we analyzed whether there exist disparities in neighborhood proximity to regulatory PM_2.5_ monitors by racial composition (%non-Hispanic White, %Black, %Hispanic, %Asian, and %American Indian or Alaskan Native (AIAN)) and socioeconomic status (operationalized as the proportion of the population living below the federal poverty line). We obtained census-tract-level demographic and socioeconomic data, including median household income, proportion of population living below the federal poverty line, and race/ethnicity in 2020 (the most recent data available) from the American Community Survey (ACS), downloaded from the Social Determinants of Health database.^50^ Census tracts with missing demographic data were excluded from the analysis, as were tracts with a total population of less than 100 to avoid noise associated with small population size (1956 tracts, 2.3%), resulting in a final sample size of 82,329 tracts. For the 2020 census, the U.S. Census Bureau defined the tracts as urban if they were within a territory that contained a densely settled core with a minimum of 2000 housing units or a population of 5000. A list of urban census tracts from the 2020 decennial census is available from the Census website.^51^ As federal guidelines and processes for selecting monitor sites differ between urban and rural counties (e.g., in rural areas, monitoring locations are chosen to capture long-range transport), all analyses were stratified by urbanicity.

To compare areas with similar high or low pollution levels, we computed census tract annual average PM_2.5_ concentrations for each year from 2019 to 2023. High-resolution PM_2.5_ estimates were obtained from the Atmospheric Analysis Composition Group’s satellite-derived PM_2.5_ data set (model *R* ^2^ = 0.67, RMSE = 2.1 μg/m^3^), created using a statistical fusion of satellite aerosol optical depth, GEOS-Chem simulation of emissions, and information from ground monitors.^25^ As satellite-derived PM_2.5_ data fall on a raster grid at a 0.01° × 0.01° resolution, we calculated the spatially weighted mean PM_2.5_ for grid cells that overlap each census tract. To reduce the impact of outliers, we computed the relative PM_2.5_ Z-score for each census tract. That is, we denoted the PM_2.5_ exposure for each tract as the number of standard deviations (SDs) from the overall mean. We used the geographic boundaries from the 2020 decennial census for all analyses to match the ACS data.

### 2.2 Statistical Analysis

We apply two statistical approaches to assess our research questions: (1) a multilevel model that allows for random effects at the county and state levels, and (2) a Bayesian multilevel model that also accounts for spatial structure in the data through the addition of a spatial error term. As tracts are clustered both by EJ attributes^52–54^ and, inherently, by monitor proximity, we expect that such spatial autocorrelation may result in biased coefficient estimates or artificially narrow confidence intervals.^55^ The Bayesian model enables us to fit a model with random effects and spatial error terms. We present the methodology and results for the nonspatial model here, as the model is simpler to interpret and both approaches yield very similar results. The method and results for the Bayesian spatial multilevel model are detailed in Appendix A. To assess the appropriateness of county- and state-level random intercepts, we calculate the proportion of variance attributable to each spatial scale by fitting a null multilevel model and dividing the variance at each level by the sum of observed variance across all levels.^56^

#### 2.2.1 Association between Census Tract EJ Attributes and Monitor Proximity

Our goal is to identify whether there are systematic associations between the EJ attributes of a census tract and proximity to the nearest monitor. Any such model should also take into account geographic differences (size, shape, and location) of each state and account for monitoring requirements that differ at a county level. As the EPA subdivides the U.S. into ten administrative regions with corresponding regional offices responsible for the execution of programs within those states, we first fit models for the U.S. as a whole and then stratify by EPA region.

To assess the association between EJ attributes and proximity to regulatory PM_2.5_ monitors across the U.S., we fit a series of multilevel models with random intercepts at the county and state levels (Equation 1). Also termed “hierarchical”^57^ or “mixed-effect”^58^ models, these are defined by the inclusion of a normally distributed random intercept (also called a “random effect”) to capture variation between groups of observations that are clustered—in our case, census tracts are clustered within counties, which in turn are clustered within states.^59^ Following the literature, we fit one model for each EJ attribute under consideration (six models for the main analysis).^47,48,60^ We consider several racial/ethnic identities because systemic racism may occur differently for different racial categories and fit separate models for each attribute to avoid overcontrolling by multiple racial/ethnic groups simultaneously. As monitoring requirements are partially informed by population and pollution levels, we controlled for population density and satellite-based annual average PM_2.5_ concentrations. The interpretation is thus that neighborhoods experiencing a similar level of pollution concentration should have monitors located, on average, at the same distance regardless of racial/ethnic composition or SES. In models with percent Black, Hispanic, Asian, or AIAN as the main predictor, we also controlled for SES and the proportion of the population identifying as White, since proportion White may or may not covary with Black, Hispanic, Asian, or AIAN populations and SES.^48,61–64^ In models where SES is the main predictor, we controlled for the proportion of non-White residents for the same reason. As a sensitivity analysis, we also consider SES and race/ethnicity independently. Equation 1 displays the model specification for *Y*_*ijk*_, the log-distance to the nearest monitor of census tract *i* in county *j* and state *k*, as a linear function of the intercept, β_0_, the EJ attribute as the main predictor, a vector of additional covariates $\vec{X}$ , with a random intercept at the county, γ_*jk*_, and state, α_*k*_, level, and residual error ϵ_*ijk*_ (see Appendix B, Section B.2.1 for model specifications). As a sensitivity analysis, we refit the same models with SES operationalized as median household income (Appendix B, Section B.2.2). All statistical analyses were conducted in R version 4.4.0.^65^

1 $$Y_{ijk}=\beta_{0}+\beta_{1}\mathrm{EJ}+\overset{⃗}{\beta_{x}\;}\vec{X}+\gamma_{jk}+\alpha_{k}+\epsilon_{ijk}$$

## 3 Results

We obtained a final sample size of 82,329 census tracts across the 48 states: 70,483 urban and 11,846 rural. Table 1 displays descriptive statistics by urbanicity. We found the average distance between the population-weighted centroid of each census tract and the nearest PM_2.5_ monitor active on at least 1 day of 2022 to be 23.5 km (SD = 28.3 km). The distance varies by urbanicity, with monitors located an average of 18.8 km (SD = 24.2 km) and 51.9 km (SD = 34.2 km) away in urban and rural census tracts, respectively. We further restricted the data to counties with at least one monitor and calculated the distance to the nearest monitor colocated within the same county as the census tract. Out of the 3107 counties included in our analysis, 612 contained at least one monitor, for a total of 55,661 census tracts (68%). For these counties, monitors were located on average 12.8 km (SD = 71.6 km) from urban and 34.4 km (SD = 137.9 km) from rural census tracts. 6170 (12%) of the 53,052 urban census tracts and 396 (15%) of the 2609 rural tracts located in a county with at least one monitor are nearer to a monitor in a neighboring county than their own.

**Table 1:** Monitor Proximity and EJ Attribute Descriptive Statistics

	Mean (SD)		
	Urban^c^	Rural^c^	All
	(*n* = 70,483)	(*n* = 11,846)	(*n* = 82,329)
Distance to nearest monitor (km)^a^			
All tracts	18.8 (24.2)	51.9 (34.2)	23.5 (28.3)
Tracts in county with monitor^b^ (*n* = 55,661)	12.8 (71.6)	34.4 (137.9)	13.8 (76.1)
EJ attributes^d^			
%AIAN	0.6 (2)	2 (10)	0.9 (4)
%Asian	6 (10)	0.6 (1.3)	5 (9)
%Black	15 (22)	7 (15)	13 (21)
%Hispanic	19 (22)	7 (13)	17 (22)
%White	58 (30)	82 (21)	61 (30)
%Below poverty	13 (11)	14 (9)	13 (11)
Median HH income (per 100,000)	0.72 (0.37)	0.57 (0.19)	0.70 (0.35)

a We stratified all statistical analyses by urbanicity.

b For each census tract, we calculated the distance from the population-weighted centroid of the tract to the nearest regulatory monitor.

c Subset of the data consisting of tracts in counties with at least one monitor.

d The mean and standard deviation (SD) of census tract EJ attributes are displayed.

The locations of PM_2.5_ monitors across the contiguous U.S. are displayed in Figure 1. The sparsity of monitoring stations—most counties contain a single monitor—results in large variability between counties both in the distance to the closest monitor, and in the association between EJ attributes and this distance. For example, consider two counties in the EPA Southeast region: Fayette County, KY, with a population of approximately 320,000, encompassing the city of Lexington (pop. 320,000), and Guilford County, NC, with a population of roughly 550,000, where Greensboro, NC, is located (pop. 300,000) (Figure 2). Each county operates exactly one PM_2.5_ monitor located within the city. Focusing on a single EJ attribute—the proportion of the census tract that identifies as Black—we see that in Fayette County, the PM_2.5_ monitor is located in and near census tracts with a large Black population, whereas in Guilford County, the opposite is true.

**Figure 1:**
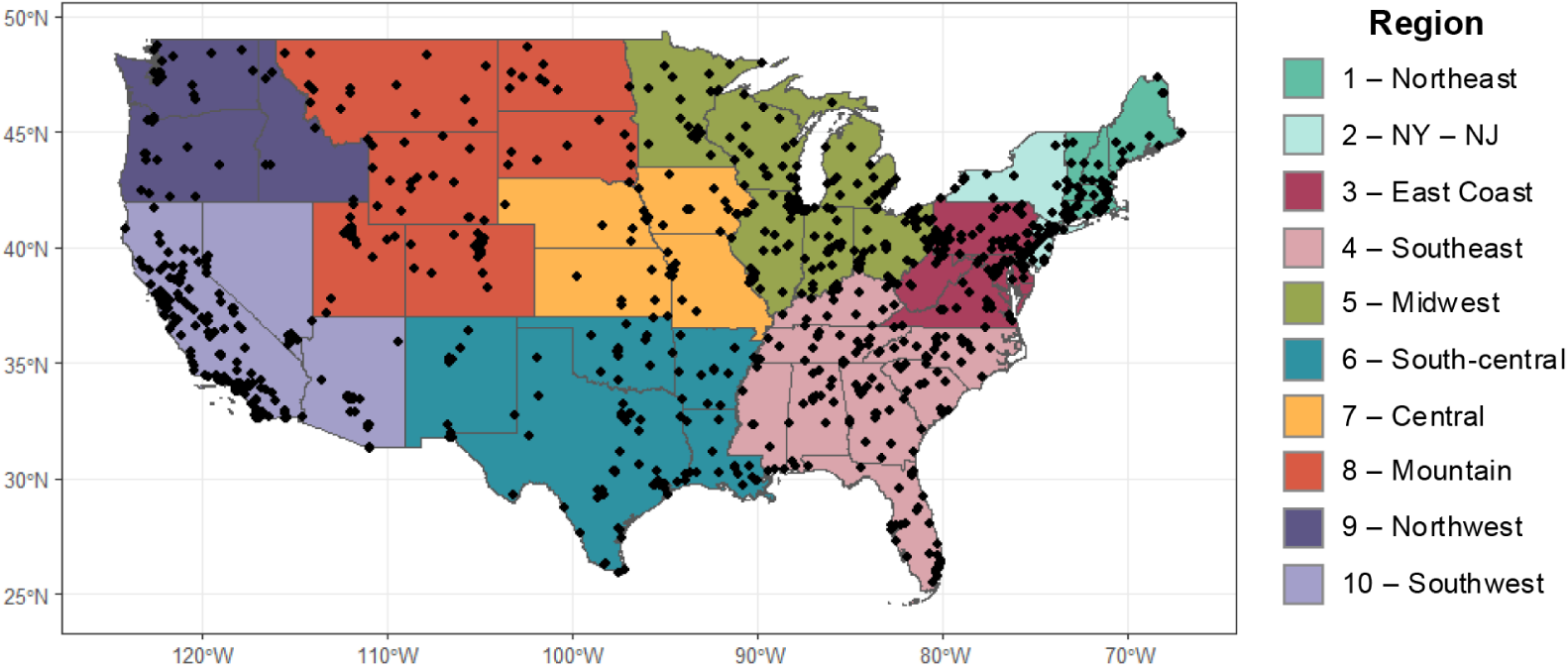
Map displaying the location of the EPA regulatory PM_2.5_ monitors (black triangles) in each state. States are grouped by EPA regions. Monitors are sparse across the U.S.: only 612 out of the 3107 counties contain at least one monitor, and monitors are located on average 18.8 km and 51.9 km from urban and rural census tracts, respectively.

**Figure 2:**
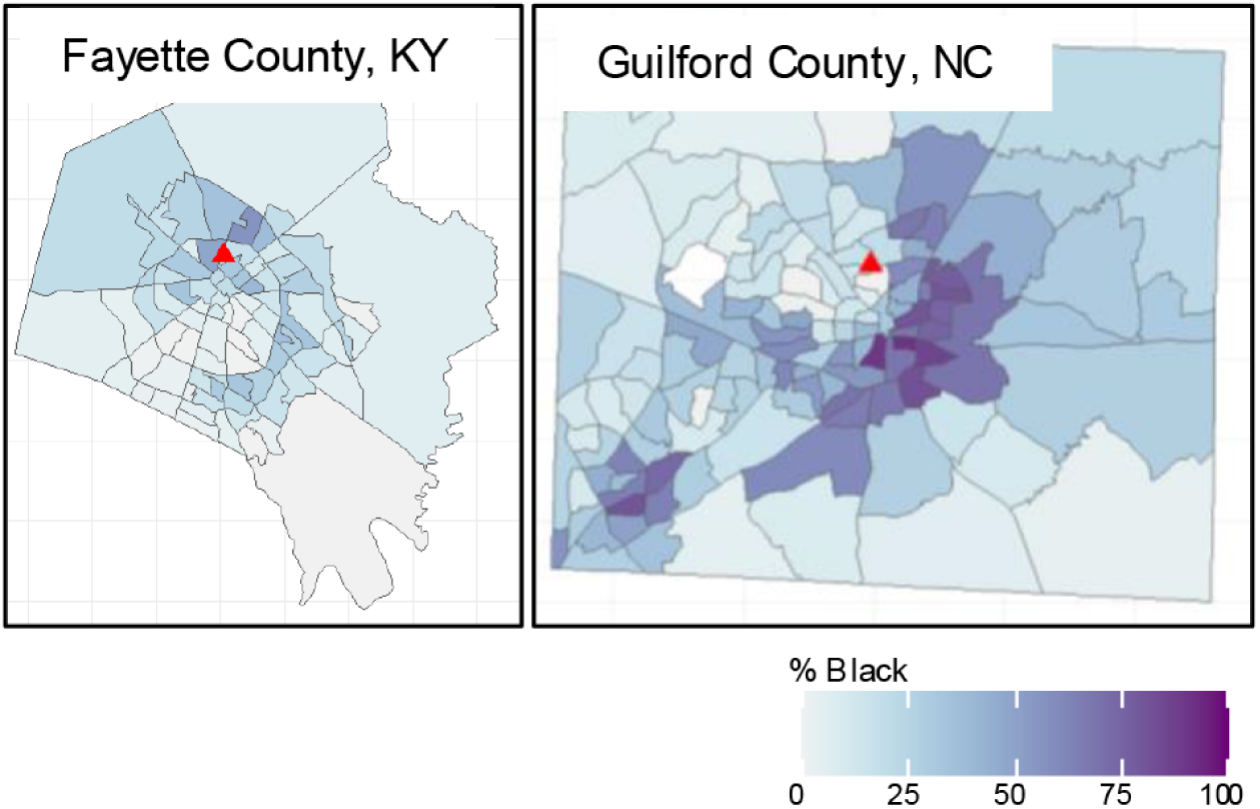
Census tracts with the percent of the population identifying as Black are displayed in blue for Lexington in Fayette County, KY, and Greensboro in Guilford County, NC. These cities each have a similar population size (320,000 and 300,000, respectively) and one regulatory PM_2.5_ monitor (denoted by the red triangles). However, in Fayette County, the PM_2.5_ monitor is located in and near census tracts with a larger Black population, while in Guilford County, the PM_2.5_ monitor is located in and near census tracts where a smaller proportion of the population is Black. With just one monitor per county, the association between the EJ attribute and proximity may vary widely between counties.

A large proportion (34%) of the nationwide variance in census tract distance to the nearest monitor site is attributable to within-county differences, even after controlling for population and urbanicity (Table S2). Yet, between-county differences remain an important source of variability, even when restricting the analysis to counties with at least one monitor, justifying the use of county-level random intercepts.

The point estimate and 95% confidence interval for the coefficients of interest in each multilevel model are presented in Figure 3. Positive coefficients indicate that the attribute is associated with a larger distance to the nearest PM_2.5_ monitor, while negative coefficients indicate a shorter distance. For the most part, the relationship between the EJ attribute and monitor proximity is weak (point estimate near zero) or insignificant (95% CI bars cross zero) for the U.S. as a whole. Among rural census tracts in the U.S.-wide model, there is a small association between a higher proportion of the population identifying as AIAN and a shorter distance to the nearest monitor (β = −0.19, 95% CI = [−0.31, −0.08]) and a higher proportion of the population identifying as Black with a longer distance (β = 0.25, 95% CI = [0.14, 0.36]) (Figure 3, U.S.-wide model results in black). Additionally, the coefficient on poverty is positive and relatively large in magnitude (β = 0.60, 95% CI = [0.50, 0.70]), indicating that having a higher proportion of the population living below the poverty line in a rural census tract is associated with a further distance to the nearest monitor. Specifically, a 10% increase in poverty is associated with a 6% increase in distance on average, all else equal (all coefficient estimates presented in Appendix B, Table S3). For example, among rural tracts with an average population density (0.004 persons/km^2^), annual PM_2.5_ concentration (Z-score = −0.78), and non-White proportion of the population (18%), we expect those with 4% of the population living below the poverty line to have the nearest monitor located 39 km away on average, as compared to 42 and 44 km for tracts with 14% poverty (the national mean %poverty for rural tracts) and 24% poverty. For rural tracts, all other EJ attribute model coefficients are weak (near-zero, |β| < 0.1) or nonstatistically significant (confidence intervals spanning zero).

**Figure 3:**
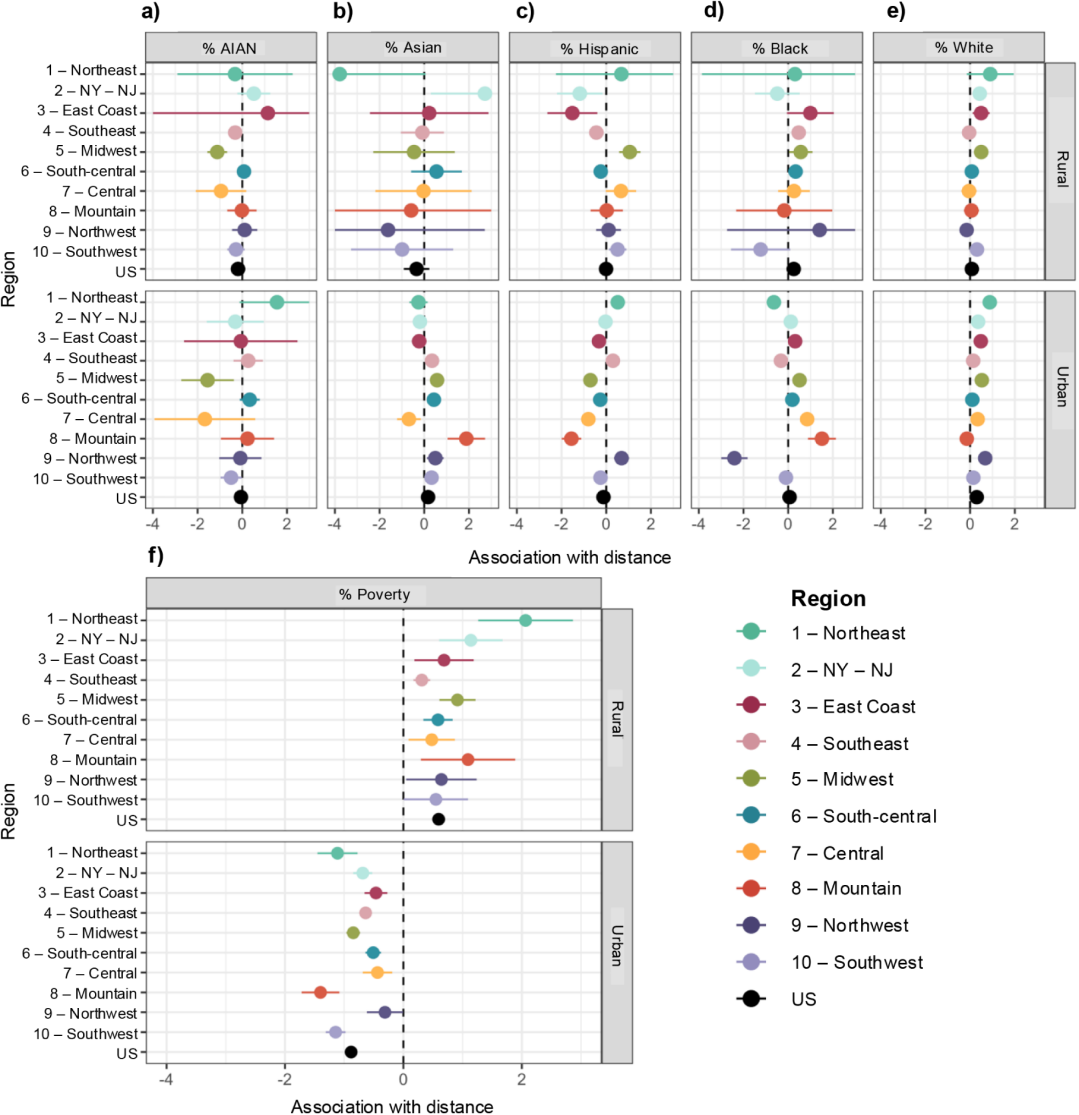
Coefficients on the association between census tract EJ attribute and distance to the nearest monitor for (a) %AIAN, (b) %Asian, (c) %Hispanic, (d) %Black, (e) %White, and (f) % living below the poverty line. The circles represent the coefficient point estimate, and the bars represent the 95% CI. Note that for some EJ attributes, the 95% CI is too narrow to display. Results from the separate models run for each U.S. EPA region are colored by region; the black point and line represent the estimate from the model for the U.S. as a whole. For the most part, the relationship between EJ attribute and monitor proximity is weak (point estimate near zero) or insignificant (95% CI bars cross zero) for the U.S. as a whole. The exception is for % poverty: for rural census tracts, a higher percent of the population living below the poverty line is significantly associated with a farther distance to the nearest monitor for all regions and the U.S. as a whole.

Conversely, for urban census tracts, higher poverty is associated with a shorter distance to the nearest monitor (β = −0.88, 95% CI = [−0.93, −0.83]). The coefficients for the proportions White and Asian are small in magnitude and positive (β = 0.30, 95% CI = [0.27, 0.33] and β = 0.17, 95% CI = [0.11, 0.23], respectively), and the coefficient on Hispanic is small in magnitude and negative (β = −0.12, 95% CI = [−0.16, −0.08]). All other EJ attributes have weak or nonstatistically significant associations (Table S4). In the sensitivity analysis, the magnitude and sign of the coefficients remain consistent when substituting the proportion of the census tract living below the poverty line for tract median household income (Figure S6, coefficients reported in Tables S5 and S6) and when considering each EJ attribute independently without additionally controlling for SES/race (Figure S7, Tables S7 and S8). In addition, coefficients on the estimates in the main models are very similar, with overlapping confidence intervals (Figure S8) across the three years considered (2019, 2022, and 2023).

Stratifying by regions, we find some heterogeneity in the coefficients of the EJ attributes (Figure 3). Specifically, for %Hispanic in rural tracts, the U.S.-wide model shows no statistically significant association overall. Yet, stratifying by region, we find a significant positive association for rural tracts in the Midwest (β = 1.06, 95% CI = [0.60, 1.55]) and a negative association for tracts in the NY-NJ region (β = −1.18, 95% CI = [−2.20, −0.16]), the East Coast (β = −1.52, 95% CI = [−2.64, −0.40]), and the Southeast (β = −0.44, 95% CI = [−0.64, −0.24]). Similarly, we find a positive association between %Hispanic and monitor proximity for urban tracts in the Northeast (β = 0.52, 95% CI = [0.23, 0.80]) and Northwest (β = 0.69, 95% CI = [0.35, 1.03]), and a negative association for tracts in the Midwest (β = −0.71, 95% CI = [−0.81, −0.61]), Central (β = −0.80, 95% CI = [−1.07, −0.54]), and Mountain regions (β = −1.56, 95% CI = [−1.99, −1.12]). We also observe some regional differences in the association between monitor proximity and %Black and %Asian for urban census tracts: most regions have near-zero values, with the exception of the Northwest (negative association β = −2.41, 95% CI = [−3.00, −1.81]) and Mountain (positive association β = 1.51, 95% CI = [0.89, 2.14]) regions for the coefficient on %Black and Mountain (positive association β = 1.88, 95% CI = [1.04, 2.72]) for %Asian (tables in Appendix B, Section B.8). For other EJ attributes, the regional subanalyses are consistent with the results of the U.S.-wide model. The coefficient on %AIAN is small and negative (Midwest) or with a confidence interval spanning zero for all regional models; the coefficient on %White is small and positive or zero for all regional models.

Examining the association between census tract poverty and PM_2.5_ regulatory monitoring, we observe a consistent and relatively strong association across all regions, with a positive association for rural and a negative association for urban census tracts. The magnitude of the association is particularly large for rural tracts in the Northeast (β = 2.06, 95% CI = [1.26, 2.86]) and urban tracts in the Northeast (β = −1.11, 95% CI = [−1.44, −0.78]), Mountain (β = −1.40, 95% CI = [−1.71, −1.09]), and Northwest (β = −1.15, 95% CI = [−1.32, −0.98]) regions.

Figure 4 displays the distribution of census tract mean PM_2.5_ concentrations for the five-year period from 2019–2023 for rural and urban tracts. While average PM_2.5_ levels are lower in rural areas (6.7 μg/m^3^) as compared to urban areas (8.1 μg/m^3^), many rural census tracts experience exposures comparable to urban areas. Disaggregating by poverty quartile shows that tracts with a higher proportion of the population living below the poverty line experience slightly higher concentrations of PM_2.5_ for both urban and rural tracts. Rural census tracts with the co-occurrence of high poverty, high PM_2.5_ levels, and greater distances to monitor (values greater than the 75th percentile for all three variables) are predominantly located in the Southeast and South-central regions. For instance, tracts in Seminole County, Georgia, and Hidalgo County, Texas, which are 150 and 72 km from the nearest monitor, respectively, have greater than 30% of the population living below the poverty line and experience annual average PM_2.5_ concentrations of 7.5 and 9 μg/m^3^.

**Figure 4:**
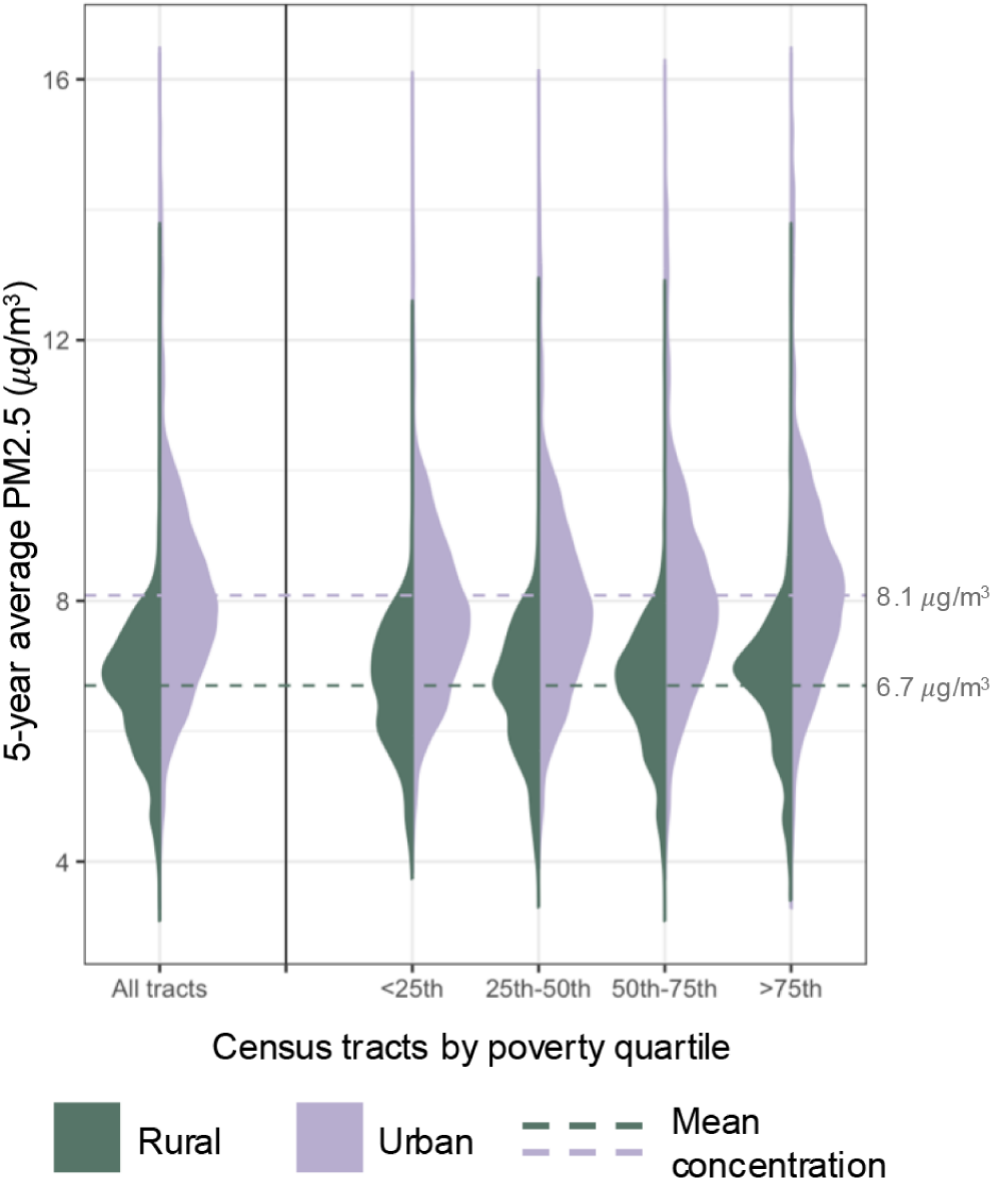
Distribution of census tracts by average PM_2.5_ concentrations (2019–2023), urbanicity, and poverty quartile. The dotted line denotes average PM_2.5_ exposure: 6.7 μg/m^3^ for rural and 8.1 μg/m^3^ for urban tracts. Many rural tracts have PM_2.5_ levels comparable to those of urban exposures.

A comparison of point estimates and 95% credible intervals shows a high degree of similarity in the estimate of associations between monitor proximity and census tract EJ attribute between the Bayesian models with and without a spatial component and strong overlap with the confidence intervals from the frequentist multilevel model (Figure S3).

## 4 Discussion

Consistent with the literature, we find that the network of regulatory PM_2.5_ monitors is sparse nationwide, with the nearest monitor located on average 18.8 and 51.9 km away from urban and rural census tracts, respectively. Consequently, the pollution levels experienced in many census tracts may not be captured by regulatory monitors. Although generally high or low concentrations of fine particles such as PM_2.5_ are considered to be a regional (e.g., state) feature,^66^ PM_2.5_ concentrations do vary substantially within cities^67^ and even within neighborhoods.^68^ Given the large within-county variance in proximity to monitors, even for counties with regulatory monitors, future work to understand which neighborhoods benefit from monitoring should be conducted at the subcounty level. Important variability in access to monitoring also exists between counties, which indicates that state-level authorities can also impact monitoring networks. Furthermore, monitor sparsity results in high heterogeneity in the association between monitor proximity and census tract EJ attributes across the country. Since census tracts are often clustered by race/ethnicity and income, randomly allocating a single monitor within a county will likely result in disparities locally, although not globally. This is not to say that monitor siting decisions within any given county or state are or are not driven by neighborhood socioeconomic and demographic composition—but that this cannot be determined from the location of the PM_2.5_ monitors alone.

We do not find systematic disparities by race/ethnicity in proximity to the nearest PM_2.5_ monitor across the U.S. This aligns with previous work by Pedde and Adar (2024),^11^ who conclude that there are little to no racial or ethnic differences in the representativeness of the covariate distribution surrounding PM_2.5_ monitors at a national level. Nevertheless, we did detect an association between monitor distance and SES, with monitors located nearer to high-poverty neighborhoods in urban areas and farther from high-poverty neighborhoods in rural areas. Similarly to Pedde and Adar (2024),^11^ we detect variability in the association between EJ attributes and monitor proximity across subregions of the U.S. This is the first study of its kind to define equity in monitoring at the neighborhood level by proximity. Related work^10^ found a statistically significant higher probability of a new NO_2_ monitor being located within a neighborhood with a higher proportion of White residents. However, this result only held for areas with low concentrations of NO_2_, and it did not test whether the difference in the probability of new monitor placement is strong enough to affect the overall network of monitors. Urban areas with higher concentrations of PM_2.5_ include locations identified in the EPA siting guidelines for monitoring (e.g., “hotspots” and significant sources of PM_2.5_) and are more likely to be near low-income communities,^69^ which may drive the association between monitor proximity and poverty in urban areas. Our results demonstrate that, regarding some attributes of concern in environmental justice research (race/ethnicity, poverty), urban EJ communities are well represented by the EPA’s regulatory PM_2.5_ monitors; the data set produced by this monitoring network does not undersample PM_2.5_ concentrations from these neighborhoods. Algorithms for estimating PM_2.5_ exposure that utilize this data set are unlikely to suffer from sampling bias by race/ethnicity or SES in urban areas.^70^

However, additional attention should be paid to monitoring in areas of rural poverty. The low density of monitors in rural areas has already been noted in the literature as impacting the accuracy of national-level gridded air pollution concentration estimates. Bechle et al. (2023)^29^ demonstrate that empirical land-use models vary more widely in their estimates of pollution in rural areas, casting doubt on the accuracy of at least some of those models (in urban areas, models largely agree). We, therefore, argue that future work constructing models for estimating PM_2.5_ should report error disaggregated by urbanicity and SES. Although PM_2.5_ concentrations are lower on average in rural as opposed to urban areas, many rural census tracts experience PM_2.5_ exposure comparable to urban or suburban neighborhoods, and disparities in exposure by race/ethnicity still exist.^2,4^ However, few previous studies have considered disparities in exposure by SES in rural areas. For instance, both Nair et al. (2023)^4^ and Tessum et al. (2021)^2^ find disparities in PM_2.5_ by race/ethnicity at a range of urbanization levels and by SES, yet do not explore the interaction of income and urbanicity. Although focused on urban areas, Knobel et al. (2023)^3^ determined that agriculture, dust, metal processing industries, and vehicle traffic sources are associated with PM_2.5_ disparities by income; other research has determined that traffic exposure disparities by income are largely driven by emissions from interstate highways.^22^ Future work is needed to assess PM_2.5_ emission sources and concentration gradients in rural areas, as these may be important drivers of exposure in low-income rural areas that are not currently surveyed by regulatory monitors.

Some limitations of the present study include the restriction of the analysis to the contiguous U.S. (without Alaska, Hawaii, or Puerto Rico, for instance). As well, there may be unmeasured covariates that are associated with monitoring site selection. We did not include information on local decision-making processes or local criteria for monitor siting beyond broad federal guidelines. Furthermore, we assume that distance to the nearest monitor can be used as a proxy to measure how well monitors capture PM_2.5_ emissions from a given census tract. This includes the implicit assumption that linear distance determines the correlation between levels of PM_2.5_ in the census tract and at the monitor, as we do not consider geographic features (e.g., mountains/ravines) or pollution transport patterns. Efforts to reduce pollution at a specific monitoring location might not necessarily lead to a decrease in pollution levels in nearby census tracts. Future work should examine the extent to which neighborhoods benefit from nearby regulatory monitors. In addition, researchers should investigate the distribution of monitors for other pollutants that are important drivers of air quality.

Despite these limitations, this study is one of the first to examine whether there exist systematic disparities in the location of regulatory PM_2.5_ monitors across the U.S. as a whole by race/ethnicity and socioeconomic status. We include tracts located both in counties with and without regulatory monitors and consider the spatial nature of the data, including the clustering of tracts by race/ethnicity and SES and by proximity to monitors. In general, we find that the network of regulatory PM_2.5_ monitors is equitably distributed, and pollution concentrations experienced by neighborhoods with EJ attributes are captured in the resulting data set. However, in rural areas, census tracts with lower SES are located farther on average from monitors than higher SES tracts after controlling for PM_2.5_ levels. Access to regulatory monitoring can be considered a key component of environmental justice. As such, efforts should be made to improve monitoring in low-income rural areas.

## Supplementary Material



## Data Availability

The monitor location data underlying this study are openly available in the Air Quality System Data Mart at https://www.epa.gov/outdoor-air-quality-data/download-daily-data. Demographic data is openly available in the Social Determinants of Health Database at https://www.ahrq.gov/sdoh/data-analytics/sdoh-data.html, and urbanicity data is openly available in the U.S. Census Bureau Block Level Urban Area information at https://www.census.gov/programs-surveys/geography/guidance/geo-areas/urban-rural.html. Satellite-derived estimates of PM_2.5_ are available from ACAG’s annual and monthly mean PM_2.5_ at https://wustl.box.com/v/ACAG-V5GL04-GWRPM25. Code is available at https://github.com/haskellcraigz/Equity_PM25_Monitors.

## Notes

Bayesian statistical modeling methods and results (Appendix A). Model specifications and coefficients (Appendix B) (PDF)
