# Supplementary material for: Equity in the Distribution of Regulatory PM2.5 Monitors
Source: Environ Sci Technol. 2025 Jul 23;59(30):15843–52. doi: 10.1021/acs.est.4c12915 (PMC12329718; doi:10.1021/acs.est.4c12915)
Supplement: Supplementary file 1 [file es4c12915_si_001.pdf]

Equity in the Distribution of Regulatory $PM_{2.5}$ Monitors

Zoé Haskell-Craig, Kevin P. Josey, Patrick L. Kinney, Priyanka deSouza

Supplementary Information

Appendix A: Bayesian multilevel model with spatial error **S2**
A.1 Methods S2
A.2 Results S7

Appendix B: Supporting Materials **S10**
B.1 Census tract $PM_{2.5}$ concentrations across the contiguous US (2019 - 2023) S10
B.2 List of multilevel models considered S11
B.3 Apportionment of variance S13
B.4 Multilevel model coefficient estimates S14
B.5 Sensitivity analysis - models with household income as SES . . . S17
B.6 Sensitivity analysis - model estimates of EJ attributes without controlling for race/poverty S20
B.7 Sensitivity Analysis - robustness of main models to year (2019, 2022 or 2023) S23
B.8 Tables with regional model coefficient estimates S24

SI References: **S37**

Appendix A Bayesian multilevel model with spatial error

Given the spatial nature of the data (census tracts are often clustered by race/ethnicity and SES [1–3] and neighboring tracts have a similar proximity to monitors), we are concerned that this structure may violate the underlying assumptions of the multilevel modelling approach employed. In particular, the assumption that the residuals are independent and identically distributed (i.i.d.), follow a normal distribution with a constant variance, and are independent of spatial location. Spatial data, however, rarely adheres to these assumptions, and model estimates may be biased and/or may underestimate the width of confidence intervals as they incorrectly treat each observation as independent [4] [5] [6]. As such, we additionally check the spatial independence of the multilevel model residuals (section A.1.2) and, if we detect spatial autocorrelation, fit a spatial model (section A.1.3).

A.1 Methods

A.1.1 Defining neighboring census tracts

To conduct any spatial analyses, we first must define which units are nearby in space (neighbors). All analysis were stratified by census tract urbanicity, resulting in two disjoint sets of census tracts with the following spatial patterns: (i) a set with "islands" of urban tracts, predominantly clustered in urban centers and non-contiguous, and (ii) a set containing a "sea" of rural tracts, separated by, and surrounding, the urban centers. As such, both sets include some census tracts with no contiguous neighbors. In order to avoid inconsistencies in how spatial models treat units with and without neighbors, we choose a definition of neighboring that does not rely on spatial contiguity and takes into account the distance between tracts. Neighbors are defined by a threshold, chosen by determining the nearest neighbor distance for each tract using kNN with $k = 1$ and then taking the threshold to be the maximum nearest distance.

To generate the spatial weight matrix $\boldsymbol{W}$, we use the inverse distance for each neighbor. That is, we calculate the distance, d, from each census tract's population-weighted centroid to all other census tracts within the threshold. Each cell of the matrix W_{ij} represents the weight for tract j on tract i and is equal to one over the distance between the two tracts for neighboring tracts and 0 otherwise:

$$w_{ij} = \begin{cases} 1/d_{ij} & \text{if } d < \text{threshold} \\ 0 & \text{otherwise} \end{cases}$$

This approach ensures that all tracts have at least one neighbor, but that distant tracts contribute relatively little importance.

Other common methods for defining neighboring census tracts rely either on spatial contiguity or consistency in the size and separation of units [7][8][9]. For instance, a common definition is the rook (tracts share a boundary edge)

or queen (tracts share an edge or a corner)-style contiguous neighbors. Computational problems arise however when there are tracts which do not share a boundary with any other tracts (islands). Typically, these tracks are simply excluded from the analysis. Another common approach is kNN. However, the literature is limited on the best approach for determining the value for k. Furthermore, the kth neighbor may be much further away for a geographically large, suburban census located on the outskirts of a densely populated area as compared to a smaller urban tract densely surrounded by other tracts. Neither a binary ($w_{ij} = 1$ for neighbors and 0 otherwise) nor a row-standardized ($w_{ij} = 1/N$, for the total number of neighbors N) encoding of neighbors into the weight matrix takes into account the distances between tracts. As an example comparison between these three methods, figures S1 and S2 display neighboring tracts for urban and rural census tracts in North Dakota and Pennsylvania. In this example we consider $k = 5$ for the kNN.

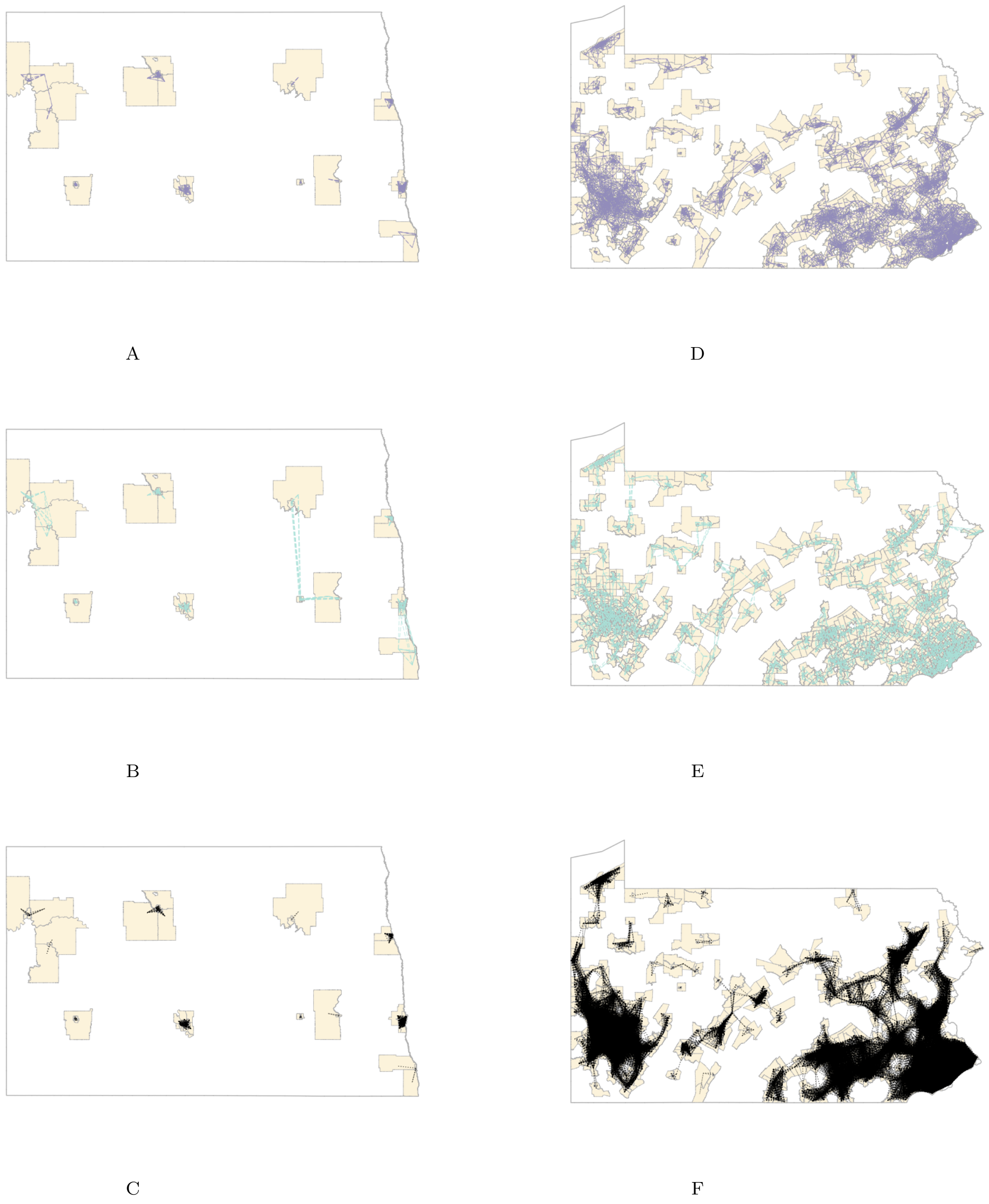

Figure S1: Cream-colored urban census tracts in North Dakota (A-C) and Pennsylvania (D-F) are separated by areas of rural tracts (not displayed). Tracts identified as neighbors are linked by purple (spatially contiguous), blue (kNN, k = 5), and black (distance-threshold) lines. Notice that kNN defines some tracts from distinct urban clusters as neighbors in North Dakota (B).

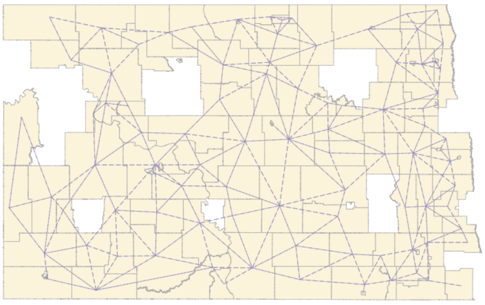

A

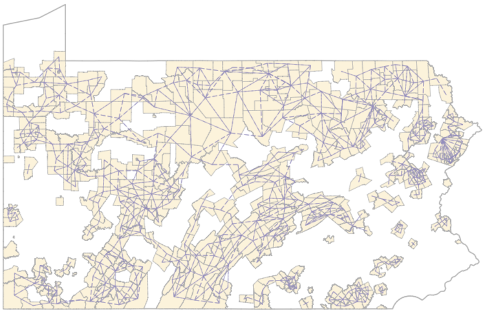

D

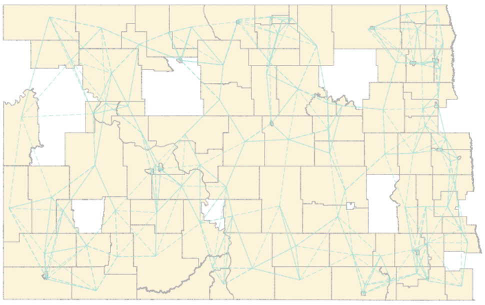

B

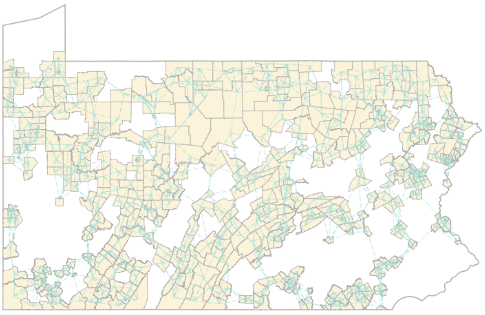

E

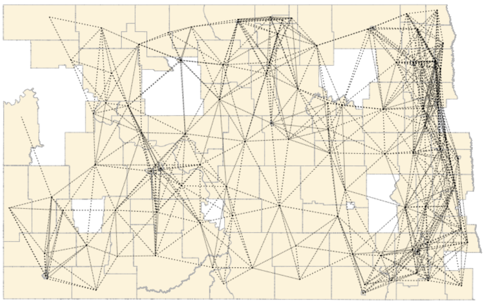

C

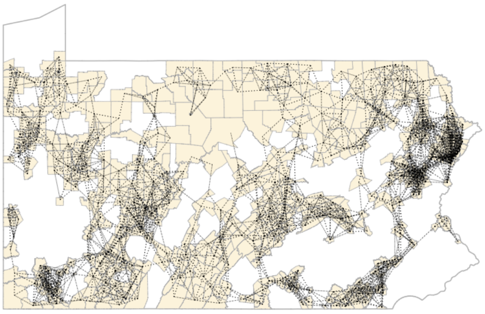

F

Figure S2: Cream-colored rural census tracts in North Dakota (A-C) and Pennsylvania (D-F). Tracts identified as neighbors are linked by purple (spatially contiguous), blue (kNN, k = 5), and black (distance-threshold) lines. Notice that the spatially contiguous definition of neighboring tracts results in some tracts with zero neighbors in Pennsylvania (D).

A.1.2 Testing for spatial autocorrelation

We assess the spatial distribution of the multilevel model residuals by performing a global Moran's I test [7] (eq. 1). The value of the Moran's I test statistic varies between -1 (negative spatial autocorrelation) and $+1$ (positive spatial autocorrelation), with a value 0 indicating no autocorrelation. A statistically significant p-value indicates the presence of spatial autocorrelation in the residuals, suggesting that the multilevel model assumption that residuals are independent is not met, and justifying the use of a spatial model [7].

$$I = \frac{n}{\sum_{i=1}^{n} \sum_{j=1}^{n} W_{ij}} \frac{\boldsymbol{\epsilon}^T \boldsymbol{W} \boldsymbol{\epsilon}}{\boldsymbol{\epsilon}^T \boldsymbol{\epsilon}}, \tag{1}$$

Here $\boldsymbol{\epsilon}$ is the vector of regression residuals from the multilevel model, and n is the total number of census tracts. $\boldsymbol{W}$ is the weight matrix defined in section A.1.1.

A.1.3 Accounting for spatial clustering

Including a random intercept at a larger spatial scale for units nested within larger geographic entities (census tracts within counties within states, for example) may or may not address the spatial dependencies in the data. We test for this residual spatial autocorrelation by applying a global Moran's I statistic to the multilevel model residuals. If statistically significant, this indicates that a spatial model may be better suited for the data.

There are two main approaches to incorporating a spatial component into a model: including a spatial "lag" term or (and) a spatial "error" term (analogous to auto-regression or moving-averages in time series analysis). However, in spatial lag models Bivand [8] cautions against the interpretation of the fitted parameters β as the expected impact of a one-unit change in the covariate on the outcome as this model (unlike the spatial error model) results in feedback. Furthermore, the implication of the spatial lag model is that the outcome for one tract depends directly on the outcome in a neighboring tract [8]. Monitor proximity is certainly correlated, yet unlike with infectious disease dynamics it does not seem realistic that proximity for one tract would be a causal mechanism driving proximity for its neighbors. For this reason, and to ensure interpretability of the coefficient on the EJ attribute variable, we choose to model the structure using a spatial error term.

To do so we take advantage of a Bayesian modelling technique that uses Integrated Nested Laplace Approximation (INLA) [10] to incorporate a spatial model component alongside random intercepts in a multilevel model [11]. INLA is a computationally efficient alternative to Markov Chain Monte Carlo (MCMC) approximations with implementations available in R through the package *R-INLA* [12]. INLA has been widely used in epidemiological studies to build multilevel spatial-temporal models with spatial error terms [13–15].

Following Tabb et al. [16], we model the county and state-level random effects as independent and identically distributed (i.i.d.) and the spatial error according to the Besag-York-Mollie (B-Y-M) model [17] (eq. 2). Similar to the

multilevel model, Y_{ijk}, the log-distance to the nearest monitor for census tract i in county j and state k, is assumed to be a linear function of fixed intercept β_0, the EJ attribute, additional covariates $\vec{X}$, and county (γ_{jk}) and state (α_k)-level random effects with an additional error term composed of a spatially structured (v_{ijk}) and a spatially unstructured (u_{ijk}) component.

$$Y_{ijk} = \beta_0 + \beta_1 EJ + \vec{\beta_x}\vec{X} + \gamma_{jk} + \alpha_k + v_{ijk} + u_{ijk} \tag{2}$$

The B-Y-M specification for the error term is a convolution of an i.i.d. Gaussian model and an intrinsic conditional autoregressive (iCAR) term [18], where the prior for unstructured error follows the usual specification of being normally distributed with a mean of 0 and constant variance σ_u^2 and the prior for the structured error, v_{ijk}, follows a normal distribution with mean m_i and variance s_i^2 [11][6] (eq. 3). Where m_i for census tract i is a function of the spatial error, v_{ljk}, of its neighboring census tracts l, and the value, w_{il} in row i and column l of the neighborhood weight matrix $\boldsymbol{W}$, defined previously. Conceptually, the interpretation of this model is that the estimates at any given location are conditional on the level of neighboring values.

$$m_i = \frac{\sum_{l=1}^{n} w_{il} v_{ljk}}{\sum_{l=1}^{n} w_{il}} \ ; \ s_i^2 = \frac{\sigma_v^2}{\sum_{l=1}^{n} w_{il}} \tag{3}$$

Random intercepts γ_{jk} and α_k are modelled as i.i.d. in *INLA*[18]. We use the default minimally informative prior of $logGamma(1, 0.0005)$ for the hyperparameter precision for all parameters (note that precision is the inverse of variance, $\tau = 1/\sigma^2$) [18]. The neighborhood matrix W was constructed using the R package *spdep* [8]. To reduce computational burden (a 70,000 x 70,000 weight matrix representing the relationship between every urban census tract in the US uses approximately 80 GB of memory), we use the *CsparseMatrix* representation from the R package *Matrix* [19] for $\boldsymbol{W}$. We retrieve the posterior mean and 95% credible intervals for fixed and random effects and compare these estimates from the Bayesian INLA model with and without a spatial error term, and to the frequentist multilevel models for each EJ attribute-model. All statistical analyses were conducted in R version 4.4.0 [20].

A.2 Results

A.2.1 Moran's I test for spatial autocorrelation in model residuals

Table S1 displays the Moran's I test statistics for spatial autocorrelation of model residuals for the US-wide multilevel models. For both urban and rural tracts, we detect a statistically significant spatial autocorrelation ($p < 0.05$) among model residuals for all EJ attributes.

Table S1: Moran's I test on US-wide multilevel model residuals.

EJ attribute model	Moran's I test results	
	Rural	Urban
%AIAN	Moran I statistic = 4.817738e-02<br>Expectation = -8.442381e-05<br>Variance = 2.363839e-06<br>p-value < 2.2e-16	Moran I statistic = 1.664633e-01<br>Expectation = -1.418802e-05<br>Variance = 3.123668e-07<br>p-value < 2.2e-16
%Asian	Moran I statistic = 4.818524e-02<br>Expectation =-8.442381e-05<br>Variance = 2.363828e-06<br>p-value < 2.2e-16	Moran I statistic = 1.664880e-01<br>Expectation = -1.418802e-05<br>Variance = 3.123668e-07<br>p-value < 2.2e-16
%Black	Moran I statistic = 4.794237e-02<br>Expectation =-8.442381e-05<br>Variance = 2.363830e-06<br>p-value < 2.2e-16	Moran I statistic = 1.661750e-01<br>Expectation = -1.418802e-05<br>Variance = 3.123668e-07<br>p-value < 2.2e-16
%Hispanic	Moran I statistic = 4.816147e-02<br>Expectation = -8.442381e-05<br>Variance = 2.363831e-06<br>p-value < 2.2e-16	Moran I statistic = 1.658527e-01<br>Expectation =-1.418802e-05<br>Variance = 3.123668e-07<br>p-value < 2.2e-16
%Poverty	Moran I statistic = 4.816533e-02<br>Expectation = -8.442381e-05<br>Variance = 2.363831e-06<br>p-value < 2.2e-16	Moran I statistic = 1.664752e-01<br>Expectation = -1.418802e-05<br>Variance = 3.123668e-07<br>p-value < 2.2e-16
%White	Moran I statistic = 4.816533e-02<br>Expectation =-8.442381e-05<br>Variance = 2.363831e-06<br>p-value < 2.2e-16	Moran I statistic = 1.664752e-01<br>Expectation = -1.418802e-05<br>Variance = 3.123668e-07<br>p-value < 2.2e-16

A.2.2 Comparison between multilevel and spatial error models

As with the non-spatial model, we construct a separate Bayesian INLA model for each EJ attribute under consideration. We compare the estimate of the coefficient on the EJ attribute for the Bayesian models with and without a spatial error term, and to the estimates from the non-spatial multilevel model (figure S3). Point estimates and 95% confidence intervals are nearly identical between all three models for all EJ attributes.

A.2.3 Comparison analysis years for spatial error models

We conduct a similar sensitivity analysis as with the non-spatial models, comparing models built with $PM_{2.5}$ concentration and monitor location data from 2022 to 2023. Coefficient estimates are robust to the year of analysis, with near-identical values and overlapping credible intervals (figure S4).

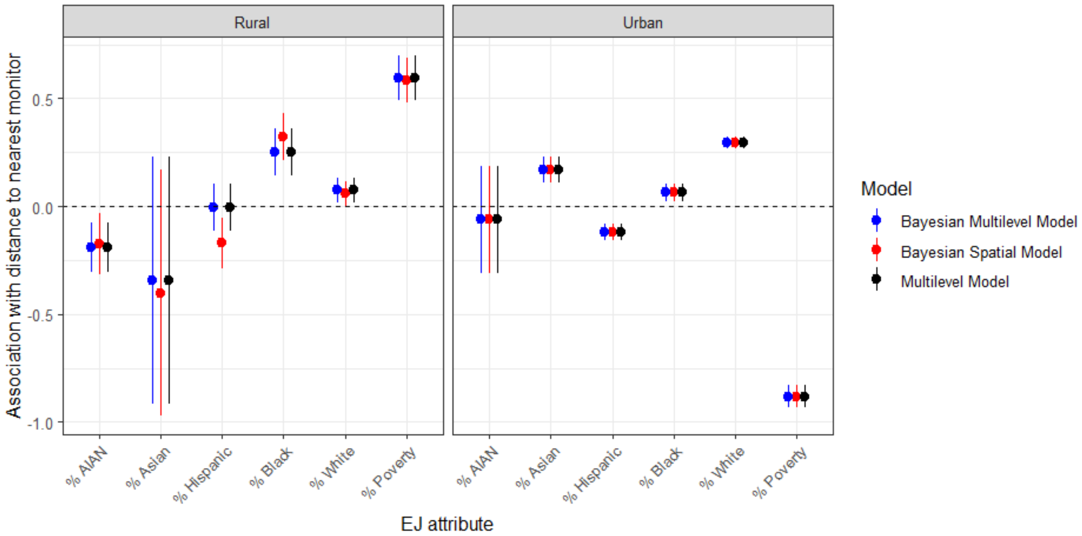

Figure S3: The association between the EJ attribute and the distance to the nearest $PM_{2.5}$ monitor for rural and urban tracts when modelled using the *INLA* Bayesian multilevel model with spatial error term (red) or without spatial error term (blue), or the frequentist multilevel model (black). Circles indicate point estimates and vertical bars the 95% credible intervals (Bayesian models) or 95% confidence intervals (frequentist model).

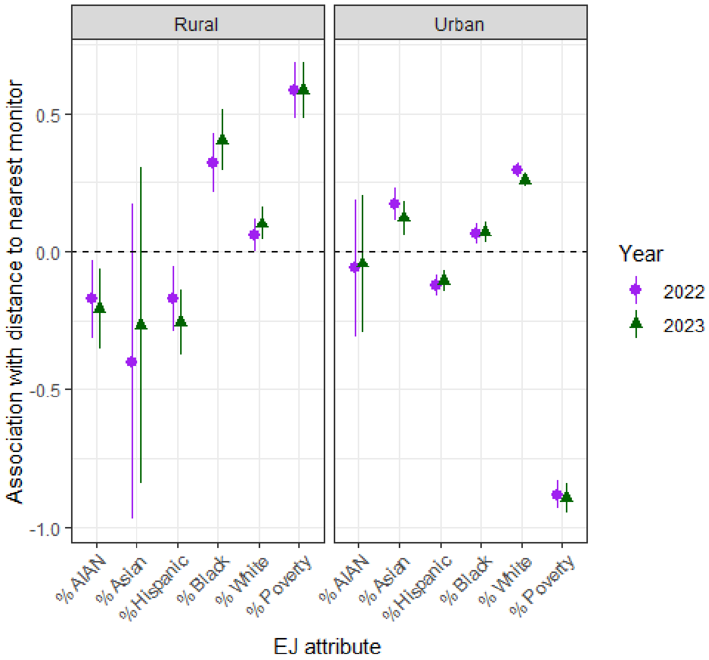

Figure S4: Estimates of the coefficient on the association between the EJ attribute and the distance to the nearest $PM_{2.5}$ monitor, comparing models constructed using data from 2022 (purple) and 2023 (green). Circles indicate point estimates and vertical bars the 95% credible intervals.

Appendix B Supporting Materials

B.1 Census tract $PM_{2.5}$ concentrations across the contiguous US (2019 - 2023)

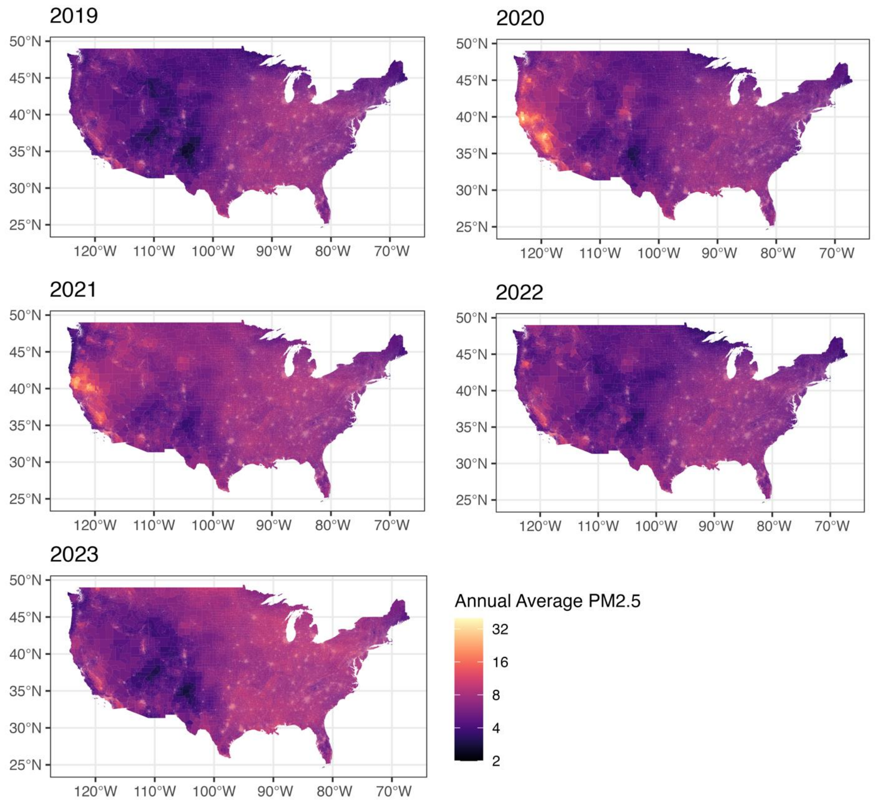

Figure S5: Annual average $PM_{2.5}$ concentrations ($\mu g/m^3$) by census tract for years 2019 - 2023.

B.2 List of multilevel models considered

All models were run separately for urban and rural census tracts.

B.2.1 Main analysis

EJ attribute: SES operationalized as the proportion of population living below the poverty line

$$\begin{aligned} Y_{ijk} = \beta_0 + \beta_1 &\text{Prop. Poverty}+ \\ &\beta_2\text{Prop non-White} + \beta_3\text{Population density} + \beta_4\text{PM2.5 Z-score}+ \\ &\gamma_{jk} + \alpha_k + \epsilon_{ijk} \end{aligned} \tag{4}$$

EJ attribute: proportion of census tract identifying as American Indian or Alaskan Native (AIAN)

$$\begin{aligned} Y_{ijk} = \beta_0 + \beta_1 &\text{Prop. AIAN}+ \\ &\beta_2\text{Prop. White} + \beta_3\text{Prop. Poverty} + \beta_4\text{Population density}+ \\ &\beta_5\text{PM2.5 Z-score} + \gamma_{jk} + \alpha_k + \epsilon_{ijk} \end{aligned} \tag{5}$$

EJ attribute: proportion of census tract identifying as Asian

$$\begin{aligned} Y_{ijk} = \beta_0 + \beta_1 &\text{Prop. Asian}+ \\ &\beta_2\text{Prop. White} + \beta_3\text{Prop. Poverty} + \beta_4\text{Population density}+ \\ &\beta_5\text{PM2.5 Z-score} + \gamma_{jk} + \alpha_k + \epsilon_{ijk} \end{aligned} \tag{6}$$

EJ attribute: proportion of census tract identifying as Black

$$\begin{aligned} Y_{ijk} = \beta_0 + \beta_1 &\text{Prop. Black}+ \\ &\beta_2\text{Prop. White} + \beta_3\text{Prop. Poverty} + \beta_4\text{Population density}+ \\ &\beta_5\text{PM2.5 Z-score} + \gamma_{jk} + \alpha_k + \epsilon_{ijk} \end{aligned} \tag{7}$$

EJ attribute: proportion of census tract identifying as Hispanic

$$\begin{aligned} Y_{ijk} = \beta_0 + \beta_1 &\text{Prop. Hispanic}+ \\ &\beta_2\text{Prop. White} + \beta_3\text{Prop. Poverty} + \beta_4\text{Population density}+ \\ &\beta_5\text{PM2.5 Z-score} + \gamma_{jk} + \alpha_k + \epsilon_{ijk} \end{aligned} \tag{8}$$

EJ attribute: proportion of census tract identifying as White

$$\begin{aligned} Y_{ijk} = \beta_0 + \beta_1 &\text{Prop. White}+ \\ &\beta_2\text{Prop. Poverty} + \beta_3\text{Population density} + \beta_4\text{PM2.5 Z-score}+ \\ &\gamma_{jk} + \alpha_k + \epsilon_{ijk} \end{aligned} \tag{9}$$

B.2.2 Sensitivity analysis: SES operationalized as median household income

EJ attribute: median household income

$$\begin{aligned} Y_{ijk} = \beta_0 + \beta_1 &\text{Household Income (in 100k)}+ \\ &\beta_2\text{Prop non-White} + \beta_3\text{Population density} + \beta_4\text{PM2.5 Z-score}+ \\ &\gamma_{jk} + \alpha_k + \epsilon_{ijk} \end{aligned} \tag{10}$$

EJ attribute: proportion of census tract identifying as American Indian or Alaskan Native (AIAN)

$$\begin{aligned}Y_{ijk} = \beta_0 + \beta_1 &\text{Prop. AIAN}+ \\ &\beta_2\text{Prop. White} + \beta_3\text{Household Income (in 100k)} + \beta_4\text{Population density}+ \\ &\beta_5\text{PM2.5 Z-score} + \gamma_{jk} + \alpha_k + \epsilon_{ijk}\end{aligned} \tag{11}$$

EJ attribute: proportion of census tract identifying as Asian

$$\begin{aligned}Y_{ijk} = \beta_0 + \beta_1 &\text{Prop. Asian}+ \\ &\beta_2\text{Prop. White} + \beta_3\text{Household Income (in 100k)} + \beta_4\text{Population density}+ \\ &\beta_5\text{PM2.5 Z-score} + \gamma_{jk} + \alpha_k + \epsilon_{ijk}\end{aligned} \tag{12}$$

EJ attribute: proportion of census tract identifying as Black

$$\begin{aligned}Y_{ijk} = \beta_0 + \beta_1 &\text{Prop. Black}+ \\ &\beta_2\text{Prop. White} + \beta_3\text{Household Income (in 100k)} + \beta_4\text{Population density}+ \\ &\beta_5\text{PM2.5 Z-score} + \gamma_{jk} + \alpha_k + \epsilon_{ijk}\end{aligned} \tag{13}$$

EJ attribute: proportion of census tract identifying as Hispanic

$$\begin{aligned}Y_{ijk} = \beta_0 + \beta_1 &\text{Prop. Hispanic}+ \\ &\beta_2\text{Prop. White} + \beta_3\text{Household Income (in 100k)} + \beta_4\text{Population density}+ \\ &\beta_5\text{PM2.5 Z-score} + \gamma_{jk} + \alpha_k + \epsilon_{ijk}\end{aligned} \tag{14}$$

EJ attribute: proportion of census tract identifying as White

$$\begin{aligned}Y_{ijk} = \beta_0 + \beta_1 &\text{Prop. White}+ \\ &\beta_2\text{Household Income (in 100k)} + \beta_3\text{Population density} + \beta_4\text{PM2.5 Z-score}+ \\ &\gamma_{jk} + \alpha_k + \epsilon_{ijk}\end{aligned} \tag{15}$$

B.3 Apportionment of variance

Table S2 displays the proportion of the total variance by state and county, before and after adjusting for census tract urbanicity and population size. We fit four multilevel linear models with random intercepts at the county and state level. Including only census tracts in counties with at least one $PM_{2.5}$ monitor, and considering proximity only to monitors located within the same county as the census tract, we fit: (i) a null model; (ii) a model adjusting for urbanicity and population. For all census tracts in the sample, considering their proximity to the nearest monitor regardless of whether this monitor was collocated within the same county or state we fit: (iii) a null model; and (iv) an adjusted model. The proportion of variance explained at the different spatial scales is calculated by dividing the variance at each level by the sum of observed variance across all scales. Across the contiguous US as a whole, the largest proportion of variance in distance to monitors (52%) can be attributed to the county-level, proceeding from the relatively small number of counties required to maintain regulatory monitors due to population size or selected for background monitoring/regional transportation sites. Within-county, that is, between-census tract residual differences remain an important source of variability (31%). When restricting the sample to census tracts located within a county with at least one monitor, the largest proportion of variability is attributed to within-county differences (67%), followed by between-county differences (27%). Controlling for census population and urbanicity does not greatly change the apportionment of variance (difference $\leq 4\%$).

Table S2: Proportion of variance attributed to each spatial scale. Variance is calculated by fitting a null multilevel model for the distance to the nearest monitor for models (i) and (iii), and a model controlling for population and urbanicity for models (ii) and (iv). We restrict the data to census tracts located in counties with at least one monitor for models (i) and (ii).

**Level**	Model i [1]	Model ii[12]	Model iii	Model iv[2]
State	0.05	0.05	0.16	0.15
County	0.27	0.24	0.52	0.51
Residual	0.67	0.71	0.31	0.34

[1] Models (i) and (ii) include only census tracts in counties containing at least one monitor

[2] Models (ii) and (iv) adjust for log(population) and urbanicity

B.4 Multilevel model coefficient estimates

Table S3: Rural

	EJ Attribute:					
	%AIAN	%Asian	%Black	%Hispanic	%Below Poverty	%White
Prop. Poverty	0.611***	0.592***	0.594***	0.595***	0.596***	0.596***
	(0.054)	(0.054)	(0.053)	(0.054)	(0.053)	(0.053)
Prop. AIAN	−0.191***					
	(0.059)					
Prop. Asian		−0.343				
		(0.292)				
Prop. Black			0.250***			
			(0.055)			
Prop. Hispanic				−0.006		
				(0.055)		
Prop. White	0.031	0.073**	0.186***	0.074**		0.075**
	(0.033)	(0.030)	(0.038)	(0.034)		(0.030)
Prop. nonWhite					−0.075**	
					(0.030)	
Pop. Density	−3.077***	−2.937***	−3.019***	−2.964***	−2.965***	−2.965***
	(0.481)	(0.481)	(0.480)	(0.480)	(0.480)	(0.480)
PM2.5 Z Score	−0.265***	−0.262***	−0.257***	−0.262***	−0.262***	−0.262***
	(0.013)	(0.013)	(0.013)	(0.013)	(0.013)	(0.013)
Intercept	10.339***	10.306***	10.201***	10.304***	10.377***	10.301***
	(0.061)	(0.060)	(0.064)	(0.063)	(0.055)	(0.060)
Observations	11,846	11,846	11,846	11,846	11,846	11,846
Note:						*p<0.1; **p<0.05; ***p<0.01

Table S4: Urban

	EJ Attribute:					
	%AIAN	%Asian	%Black	%Hispanic	% Below Poverty	%White
Prop. Poverty	-0.882^{***}	-0.868^{***}	-0.892^{***}	-0.891^{***}	-0.883^{***}	-0.883^{***}
	(0.027)	(0.027)	(0.027)	(0.027)	(0.027)	(0.027)
Prop. AIAN	-0.061					
	(0.126)					
Prop. Asian		0.171^{***}				
		(0.031)				
Prop. Black			0.064^{***}			
			(0.020)			
Prop. Hispanic				-0.121^{***}		
				(0.019)		
Prop. White	0.295^{***}	0.304^{***}	0.331^{***}	0.249^{***}		0.296^{***}
	(0.013)	(0.013)	(0.017)	(0.015)		(0.013)
Prop. nonWhite					-0.296^{***}	
					(0.013)	
Pop. Density	-0.197^{***}	-0.198^{***}	-0.194^{***}	-0.191^{***}	-0.197^{***}	-0.197^{***}
	(0.008)	(0.008)	(0.008)	(0.008)	(0.008)	(0.008)
PM2.5 Z Score	-0.320^{***}	-0.319^{***}	-0.317^{***}	-0.315^{***}	-0.319^{***}	-0.319^{***}
	(0.006)	(0.006)	(0.006)	(0.006)	(0.006)	(0.006)
Intercept	9.811^{***}	9.798^{***}	9.780^{***}	9.861^{***}	10.105^{***}	9.810^{***}
	(0.078)	(0.078)	(0.079)	(0.079)	(0.078)	(0.078)
Observations	70,483	70,483	70,483	70,483	70,483	70,483

Note: *p<0.1; **p<0.05; ***p<0.01

B.5 Sensitivity analysis - models with household income as SES

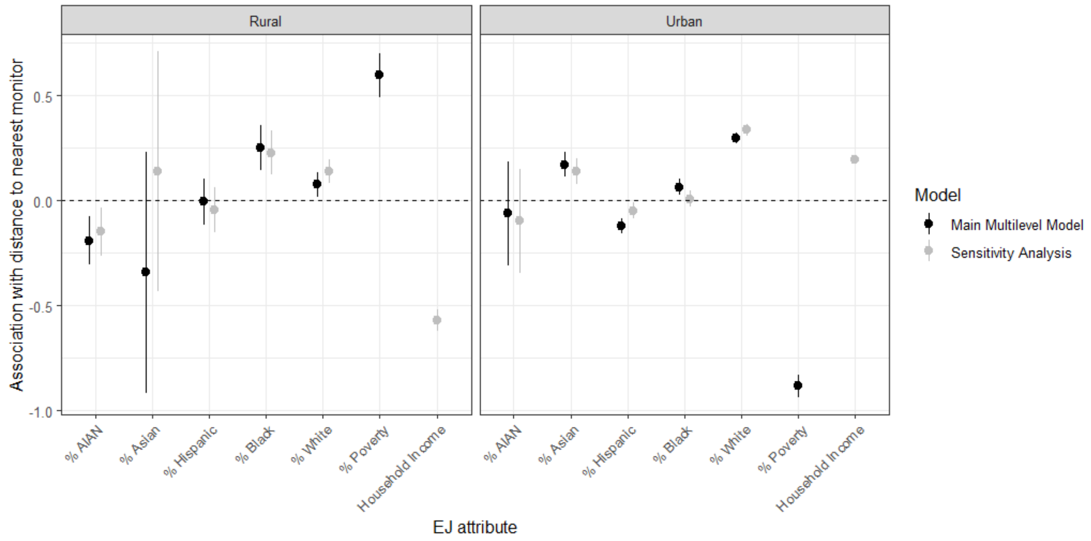

Figure S6: The association between the EJ attribute and the distance to the nearest $PM_{2.5}$ monitor for rural and urban tracts. A comparison can be seen between the estimate of the EJ coefficient in models with SES operationalized as % poverty (black) as in the main models and median household income (grey) in the sensitivity analysis. Circles indicate point estimates and vertical bars the 95% confidence intervals. Coefficients are very similar with largely overlapping 95% confidence intervals. Note that a higher value of % poverty indicates a tract with lower SES whereas a higher value of median household income indicates higher SES.

Table S5: Sensitivity Analysis: Household Income (Rural)

	EJ Attribute:					
	%AIAN	%Asian	%Black	%Hispanic	HH Income	%White
Median HH Income (100k)	−0.571***	−0.571***	−0.567***	−0.570***	−0.570***	−0.570***
	(0.027)	(0.027)	(0.027)	(0.027)	(0.027)	(0.027)
Prop. AIAN	−0.149**					
	(0.058)					
Prop. Asian		0.138				
		(0.290)				
Prop. Black			0.228***			
			(0.054)			
Prop. Hispanic				−0.044		
				(0.054)		
Prop. White	0.100***	0.138***	0.237***	0.124***		0.137***
	(0.032)	(0.029)	(0.038)	(0.033)		(0.029)
Prop. nonWhite					−0.137***	
					(0.029)	
Pop. Density	−3.329***	−3.260***	−3.293***	−3.239***	−3.247***	−3.247***
	(0.477)	(0.477)	(0.476)	(0.476)	(0.476)	(0.476)
PM2.5 Z Score	−0.255***	−0.253***	−0.249***	−0.251***	−0.253***	−0.253***
	(0.013)	(0.013)	(0.013)	(0.013)	(0.013)	(0.013)
Intercept	10.716***	10.683***	10.591***	10.698***	10.820***	10.684***
	(0.056)	(0.054)	(0.059)	(0.057)	(0.052)	(0.054)
Observations	11,846	11,846	11,846	11,846	11,846	11,846

Note: *p<0.1; **p<0.05; ***p<0.01

Table S6: Sensitivity Analysis: Household income (Urban)

	EJ Attribute:					
	%AIAN	%Asian	%Black	%Hispanic	HH Income	%White
Median HH Income (100k)	0.195***	0.186***	0.195***	0.193***	0.195***	0.195***
	(0.009)	(0.010)	(0.009)	(0.009)	(0.009)	(0.009)
Prop. AIAN	−0.098					
	(0.127)					
Prop. Asian		0.139***				
		(0.032)				
Prop. Black			0.007			
			(0.020)			
Prop. Hispanic				−0.048**		
				(0.019)		
Prop. White	0.334***	0.346***	0.339***	0.319***		0.335***
	(0.014)	(0.014)	(0.018)	(0.015)		(0.013)
Prop. nonWhite					−0.335***	
					(0.013)	
Pop. Density	−0.206***	−0.207***	−0.205***	−0.204***	−0.206***	−0.206***
	(0.008)	(0.008)	(0.008)	(0.008)	(0.008)	(0.008)
PM2.5 Z Score	−0.315***	−0.315***	−0.314***	−0.313***	−0.315***	−0.315***
	(0.006)	(0.006)	(0.006)	(0.006)	(0.006)	(0.006)
Intercept	9.541***	9.533***	9.535***	9.558***	9.874***	9.539***
	(0.078)	(0.078)	(0.078)	(0.078)	(0.078)	(0.078)
Observations	70,483	70,483	70,483	70,483	70,483	70,483
Note:						*p<0.1; **p<0.05; ***p<0.01

B.6 Sensitivity analysis - model estimates of EJ attributes without controlling for race/poverty

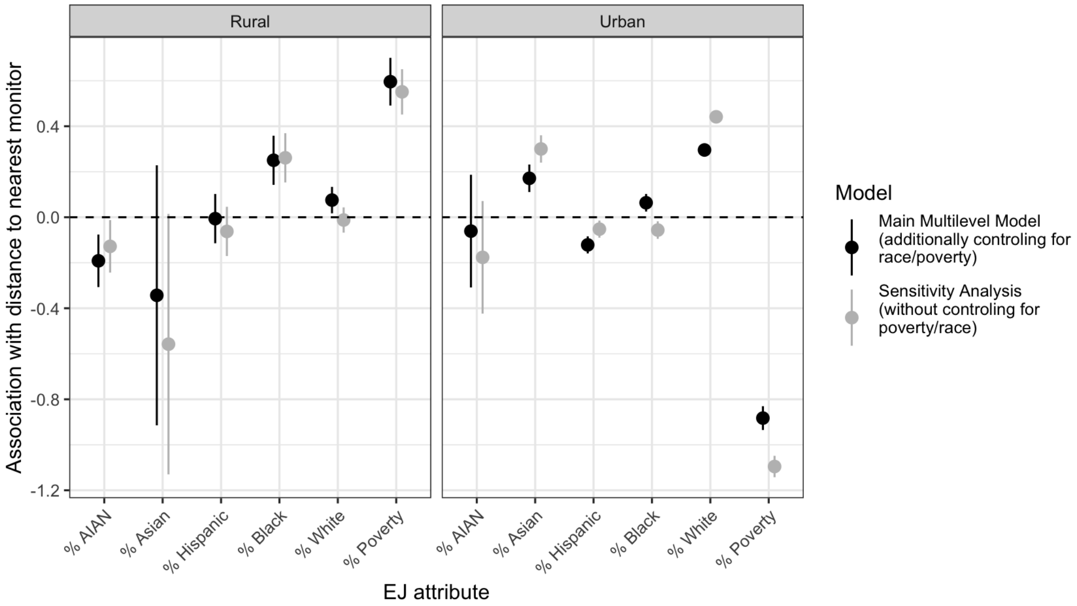

Figure S7: The association between the EJ attribute and the distance to the nearest $PM_{2.5}$ monitor when models for race/ethnicity control for %poverty (Main MLM - in black) and when EJ attributes are considered independently (Sensitivity Analysis - in grey) (i.e. models for race/ethnicity do not control for %poverty and models for %poverty do not control for race/ethnicity). Circles indicate point estimates and vertical bars the 95% confidence intervals. For rural tracts coefficients are very similar with overlapping 95% confidence intervals. In urban areas a slightly stronger association is detected for % White in models without the additional control, suggesting that the association is partially mediated by %Poverty (and likewise for %Poverty).

Table S7: Sensitivity Analysis: EJ attributes w/o additionally controlling for race/poverty (Rural)

	EJ Attribute:					
	% AIAN	% Asian	% Black	% Hispanic	% Poverty	% White
Prop. AIAN	−0.128**					
	(0.059)					
Prop. Asian		−0.558*				
		(0.292)				
Prop. Black			0.261***			
			(0.055)			
Prop. Hispanic				−0.062		
				(0.055)		
Prop. White	−0.044	−0.015	0.103***	−0.030		−0.012
	(0.032)	(0.028)	(0.037)	(0.032)		(0.028)
Prop. Poverty					0.551***	
					(0.051)	
Pop. Density	−2.875***	−2.761***	−2.856***	−2.789***	−3.195***	−2.804***
	(0.481)	(0.481)	(0.480)	(0.480)	(0.478)	(0.480)
PM2.5 Z Score	−0.293***	−0.291***	−0.286***	−0.289***	−0.291***	−0.291***
	(0.014)	(0.014)	(0.014)	(0.014)	(0.014)	(0.014)
Intercept	10.440***	10.418***	10.308***	10.433***	10.334***	10.412***
	(0.062)	(0.060)	(0.065)	(0.063)	(0.055)	(0.060)
Observations	11,846	11,846	11,846	11,846	11,846	11,846
Note:						*p<0.1; **p<0.05; ***p<0.01

Table S8: Sensitivity Analysis: EJ attributes w/o additionally controlling for race/poverty (Urban)

	EJ Attribute:					
	% AIAN	% Asian	% Black	% Hispanic	% Poverty	% White
Prop. AIAN	−0.176					
	(0.126)					
Prop. Asian		0.300***				
		(0.031)				
Prop. Black			−0.056***			
			(0.020)			
Prop. Hispanic				−0.052***		
				(0.019)		
Prop. White	0.440***	0.451***	0.408***	0.422***		0.441***
	(0.012)	(0.012)	(0.016)	(0.014)		(0.012)
Prop. Poverty					−1.095***	
					(0.024)	
Pop. Density	−0.209***	−0.209***	−0.212***	−0.207***	−0.187***	−0.209***
	(0.008)	(0.008)	(0.008)	(0.008)	(0.008)	(0.008)
PM2.5 Z Score	−0.398***	−0.398***	−0.400***	−0.396***	−0.413***	−0.398***
	(0.006)	(0.006)	(0.006)	(0.006)	(0.006)	(0.006)
Intercept	9.516***	9.499***	9.541***	9.534***	9.986***	9.512***
	(0.079)	(0.079)	(0.080)	(0.079)	(0.079)	(0.079)
Observations	70,483	70,483	70,483	70,483	70,483	70,483

Note: *p<0.1; **p<0.05; ***p<0.01

B.7 Sensitivity Analysis - robustness of main models to year (2019, 2022 or 2023)

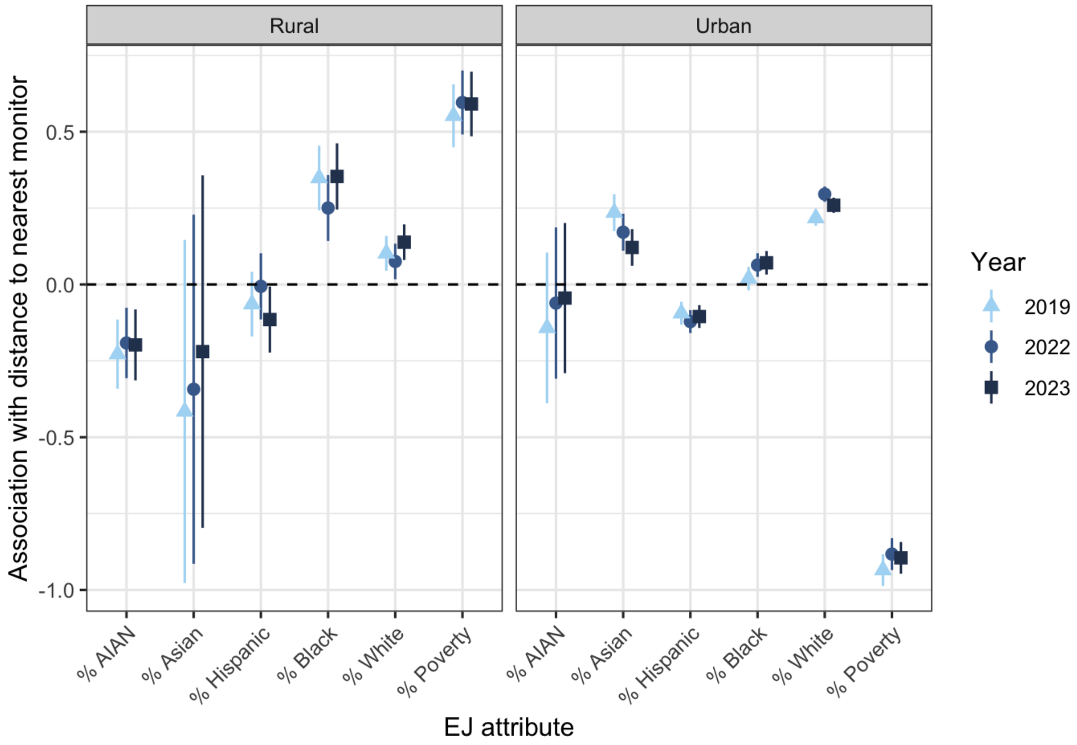

Figure S8: Comparison of the estimates of the coefficient on EJ attributes for models constructed with monitor site location and satellite-based $PM_{2.5}$ data for the years 2019, 2022, and 2023. Coefficient estimates are robust to the year of analysis (very similar point estimates and 95% CIs). Note that demographic data is from the 2020 ACS for all models.

B.8 Tables with regional model coefficient estimates

Table S9: EJ Attribute: %AIAN (Rural)

	EPA Region									
	(1)	(2)	(3)	(4)	(5)	(6)	(7)	(8)	(9)	(10)
Prop. Poverty	2.070***	1.173***	0.688***	0.308***	0.957***	0.575***	0.482**	1.097***	0.624**	0.621**
	(0.409)	(0.274)	(0.256)	(0.072)	(0.155)	(0.127)	(0.199)	(0.419)	(0.307)	(0.282)
Prop. White	0.824	0.699***	0.502**	−0.058	0.053	0.083	−0.157	0.046	−0.103	0.164
	(0.621)	(0.252)	(0.198)	(0.042)	(0.138)	(0.066)	(0.186)	(0.305)	(0.209)	(0.170)
Prop. AIAN	−0.325	0.517	1.145	−0.317**	−1.121***	0.076	−0.952*	−0.017	0.107	−0.284
	(1.314)	(0.375)	(4.024)	(0.158)	(0.225)	(0.131)	(0.575)	(0.336)	(0.288)	(0.199)
Pop. Density	−12.536**	−1.883	−1.311	−5.160***	−2.083**	−2.583*	−3.733	−1.589	−4.223	−7.938***
	(6.022)	(1.205)	(1.058)	(1.374)	(0.994)	(1.319)	(2.528)	(3.615)	(3.932)	(2.719)
PM2.5 Z Score	−0.252**	−0.144	−0.264***	−0.373***	−0.317***	−0.374***	−0.491***	−0.107	−0.298***	−0.121***
	(0.116)	(0.118)	(0.080)	(0.033)	(0.035)	(0.033)	(0.058)	(0.085)	(0.042)	(0.038)
Intercept	8.945***	9.349***	9.581***	10.482***	10.238***	10.549***	10.766***	10.650***	10.562***	10.569***
	(0.622)	(0.506)	(0.220)	(0.105)	(0.145)	(0.083)	(0.209)	(0.311)	(0.209)	(0.375)
Observations	485	526	779	3,195	2,206	1,742	1,001	592	410	517

*p<0.1; **p<0.05; ***p<0.01

Table S10: EJ Attribute: %AIAN (Urban)

	EPA Region									
	(1)	(2)	(3)	(4)	(5)	(6)	(7)	(8)	(9)	(10)
Prop. Poverty	−1.113***	−0.685***	−0.463***	−0.638***	−0.845***	−0.512***	−0.428***	−1.408***	−0.310**	−1.133***
	(0.173)	(0.082)	(0.098)	(0.054)	(0.059)	(0.067)	(0.127)	(0.164)	(0.156)	(0.085)
Prop. White	0.896***	0.354***	0.477***	0.138***	0.518***	0.101***	0.329***	−0.141	0.672***	0.134***
	(0.076)	(0.033)	(0.047)	(0.027)	(0.030)	(0.034)	(0.067)	(0.099)	(0.093)	(0.036)
Prop. AIAN	1.555*	−0.313	−0.067	0.264	−1.555***	0.332	−1.676	0.233	−0.087	−0.500**
	(0.858)	(0.651)	(1.296)	(0.335)	(0.600)	(0.229)	(1.150)	(0.608)	(0.481)	(0.242)
Pop. Density	−0.519***	−0.093***	−0.313***	−0.636***	−0.243***	−0.704***	−1.974***	−0.734***	−0.322***	−0.122***
	(0.055)	(0.010)	(0.039)	(0.038)	(0.027)	(0.053)	(0.142)	(0.123)	(0.062)	(0.025)
PM2.5 Z Score	−0.888***	−0.496***	−0.579***	−0.383***	−0.593***	−0.256***	−0.607***	−0.527***	−0.776***	−0.239***
	(0.056)	(0.027)	(0.026)	(0.016)	(0.025)	(0.018)	(0.050)	(0.049)	(0.034)	(0.009)
Intercept	7.854***	9.152***	9.143***	10.181***	9.655***	10.477***	10.108***	9.538***	9.421***	9.911***
	(0.178)	(0.430)	(0.165)	(0.086)	(0.132)	(0.094)	(0.237)	(0.214)	(0.354)	(0.410)
Observations	3,151	6,839	5,057	13,792	11,746	8,985	2,900	2,487	2,793	10,975

*p<0.1; **p<0.05; ***p<0.01

Table S11: EJ Attribute: %Asian (Rural)

	EPA Region									
	(1)	(2)	(3)	(4)	(5)	(6)	(7)	(8)	(9)	(10)
Prop. Poverty	1.937***	1.210***	0.687***	0.311***	0.908***	0.589***	0.480**	1.076***	0.632**	0.525*
	(0.412)	(0.274)	(0.256)	(0.072)	(0.156)	(0.126)	(0.200)	(0.411)	(0.304)	(0.279)
Prop. White	0.455	0.566***	0.501**	−0.044	0.488***	0.076	−0.051	0.051	−0.157	0.305**
	(0.582)	(0.170)	(0.202)	(0.041)	(0.107)	(0.063)	(0.178)	(0.174)	(0.156)	(0.138)
Prop. Asian	−3.783*	2.717**	0.220	−0.084	−0.457	0.549	−0.035	−0.585	−1.621	−0.991
	(1.961)	(1.235)	(1.353)	(0.492)	(0.931)	(0.578)	(1.099)	(1.991)	(2.210)	(1.166)
Pop. Density	−12.135**	−2.386**	−1.317	−5.040***	−1.502	−2.686**	−3.372	−1.381	−4.161	−7.577***
	(5.962)	(1.204)	(1.058)	(1.375)	(0.996)	(1.312)	(2.519)	(3.648)	(3.927)	(2.714)
PM2.5 Z Score	−0.239**	−0.139	−0.263***	−0.375***	−0.310***	−0.376***	−0.488***	−0.106	−0.300***	−0.105***
	(0.116)	(0.117)	(0.080)	(0.033)	(0.035)	(0.033)	(0.058)	(0.085)	(0.041)	(0.036)
Intercept	9.348***	9.447***	9.582***	10.468***	9.832***	10.553***	10.663***	10.652***	10.618***	10.490***
	(0.598)	(0.480)	(0.224)	(0.105)	(0.122)	(0.082)	(0.203)	(0.225)	(0.178)	(0.362)
Observations	485	526	779	3,195	2,206	1,742	1,001	592	410	517

*p<0.1; **p<0.05; ***p<0.01

Table S12: EJ Attribute: %Asian (Urban)

	EPA Region									
	(1)	(2)	(3)	(4)	(5)	(6)	(7)	(8)	(9)	(10)
Prop. Poverty	−1.120***	−0.703***	−0.474***	−0.619***	−0.823***	−0.469***	−0.447***	−1.429***	−0.246	−1.070***
	(0.173)	(0.082)	(0.099)	(0.054)	(0.059)	(0.068)	(0.127)	(0.162)	(0.157)	(0.086)
Prop. White	0.869***	0.342***	0.475***	0.138***	0.535***	0.102***	0.324***	−0.087	0.805***	0.196***
	(0.075)	(0.034)	(0.047)	(0.027)	(0.029)	(0.034)	(0.066)	(0.098)	(0.104)	(0.037)
Prop. Asian	−0.257	−0.195***	−0.228	0.348***	0.574***	0.432***	−0.686**	1.884***	0.496***	0.328***
	(0.209)	(0.062)	(0.160)	(0.115)	(0.093)	(0.096)	(0.270)	(0.428)	(0.191)	(0.058)
Pop. Density	−0.516***	−0.094***	−0.308***	−0.641***	−0.260***	−0.725***	−1.933***	−0.746***	−0.346***	−0.119***
	(0.055)	(0.010)	(0.039)	(0.038)	(0.027)	(0.053)	(0.143)	(0.122)	(0.062)	(0.025)
PM2.5 Z Score	−0.883***	−0.507***	−0.579***	−0.388***	−0.594***	−0.264***	−0.603***	−0.534***	−0.770***	−0.234***
	(0.056)	(0.027)	(0.026)	(0.016)	(0.025)	(0.018)	(0.050)	(0.049)	(0.034)	(0.009)
Intercept	7.896***	9.163***	9.150***	10.173***	9.619***	10.474***	10.114***	9.468***	9.306***	9.838***
	(0.177)	(0.425)	(0.165)	(0.086)	(0.135)	(0.091)	(0.237)	(0.211)	(0.356)	(0.411)
Observations	3,151	6,839	5,057	13,792	11,746	8,985	2,900	2,487	2,793	10,975

*p<0.1; **p<0.05; ***p<0.01

Table S13: EJ Attribute: %Black (Rural)

	EPA Region									
	(1)	(2)	(3)	(4)	(5)	(6)	(7)	(8)	(9)	(10)
Prop. Poverty	2.059***	1.152***	0.679***	0.300***	0.919***	0.557***	0.469**	1.086***	0.647**	0.572**
	(0.410)	(0.273)	(0.256)	(0.072)	(0.155)	(0.126)	(0.200)	(0.409)	(0.304)	(0.277)
Prop. White	0.933	0.349*	1.060***	0.340***	0.614***	0.184**	0.024	0.053	−0.154	0.260*
	(0.580)	(0.180)	(0.365)	(0.082)	(0.120)	(0.074)	(0.203)	(0.175)	(0.156)	(0.140)
Prop. Black	0.305	−0.488	0.993*	0.469***	0.563**	0.333***	0.259	−0.176	1.410	−1.232*
	(2.124)	(0.509)	(0.535)	(0.087)	(0.268)	(0.119)	(0.362)	(1.100)	(2.115)	(0.676)
Pop. Density	−12.504**	−2.103*	−1.247	−5.066***	−1.573	−2.786**	−3.460	−1.512	−4.351	−7.284***
	(6.056)	(1.201)	(1.057)	(1.369)	(0.993)	(1.310)	(2.522)	(3.608)	(3.918)	(2.710)
PM2.5 Z Score	−0.252**	−0.128	−0.281***	−0.360***	−0.313***	−0.380***	−0.492***	−0.106	−0.301***	−0.106***
	(0.116)	(0.117)	(0.081)	(0.033)	(0.035)	(0.033)	(0.058)	(0.085)	(0.041)	(0.036)
Intercept	8.839***	9.698***	9.014***	10.104***	9.704***	10.458***	10.590***	10.647***	10.594***	10.519***
	(0.592)	(0.480)	(0.379)	(0.122)	(0.133)	(0.091)	(0.223)	(0.225)	(0.180)	(0.368)
Observations	485	526	779	3,195	2,206	1,742	1,001	592	410	517

*p<0.1; **p<0.05; ***p<0.01

Table S14: EJ Attribute: %Black (Urban)

	EPA Region									
	(1)	(2)	(3)	(4)	(5)	(6)	(7)	(8)	(9)	(10)
Prop. Poverty	−1.158***	−0.680***	−0.459***	−0.585***	−0.939***	−0.530***	−0.441***	−1.401***	−0.221	−1.134***
	(0.173)	(0.082)	(0.098)	(0.054)	(0.059)	(0.068)	(0.126)	(0.162)	(0.154)	(0.086)
Prop. White	0.600***	0.431***	0.729***	−0.112***	0.913***	0.186***	0.971***	0.092	0.291***	0.137***
	(0.103)	(0.045)	(0.081)	(0.043)	(0.046)	(0.041)	(0.112)	(0.109)	(0.103)	(0.037)
Prop. Black	−0.639***	0.117**	0.312***	−0.324***	0.510***	0.185***	0.850***	1.512***	−2.406***	−0.098
	(0.164)	(0.047)	(0.081)	(0.044)	(0.046)	(0.050)	(0.121)	(0.319)	(0.302)	(0.095)
Pop. Density	−0.552***	−0.089***	−0.277***	−0.665***	−0.195***	−0.688***	−1.779***	−0.754***	−0.349***	−0.122***
	(0.056)	(0.010)	(0.040)	(0.038)	(0.027)	(0.053)	(0.143)	(0.122)	(0.061)	(0.025)
PM2.5 Z Score	−0.893***	−0.493***	−0.569***	−0.394***	−0.563***	−0.251***	−0.601***	−0.522***	−0.761***	−0.237***
	(0.056)	(0.027)	(0.026)	(0.016)	(0.025)	(0.018)	(0.049)	(0.049)	(0.033)	(0.009)
Intercept	8.134***	9.088***	8.906***	10.413***	9.303***	10.414***	9.537***	9.339***	9.741***	9.897***
	(0.187)	(0.427)	(0.177)	(0.094)	(0.131)	(0.103)	(0.240)	(0.216)	(0.355)	(0.405)
Observations	3,151	6,839	5,057	13,792	11,746	8,985	2,900	2,487	2,793	10,975

*p<0.1; **p<0.05; ***p<0.01

Table S15: EJ Attribute: %Hispanic (Rural)

	EPA Region									
	(1)	(2)	(3)	(4)	(5)	(6)	(7)	(8)	(9)	(10)
Prop. Poverty	2.078***	1.184***	0.667***	0.307***	0.931***	0.542***	0.501**	1.097***	0.661**	0.671**
	(0.409)	(0.273)	(0.256)	(0.072)	(0.155)	(0.127)	(0.200)	(0.415)	(0.308)	(0.280)
Prop. White	1.065*	0.235	0.024	−0.097**	0.780***	−0.057	0.275	0.063	−0.103	0.544***
	(0.645)	(0.180)	(0.260)	(0.043)	(0.125)	(0.082)	(0.244)	(0.190)	(0.211)	(0.166)
Prop. Hispanic	0.679	−1.181**	−1.516***	−0.442***	1.055***	−0.246**	0.661*	0.022	0.105	0.511**
	(1.494)	(0.520)	(0.571)	(0.102)	(0.245)	(0.098)	(0.347)	(0.369)	(0.283)	(0.200)
Pop. Density	−12.527**	−1.823	−1.397	−4.957***	−2.034**	−2.553*	−3.366	−1.583	−4.467	−8.151***
	(5.994)	(1.200)	(1.055)	(1.371)	(0.997)	(1.310)	(2.516)	(3.600)	(3.933)	(2.703)
PM2.5 Z Score	−0.254**	−0.163	−0.276***	−0.366***	−0.315***	−0.376***	−0.478***	−0.107	−0.304***	−0.138***
	(0.116)	(0.118)	(0.080)	(0.033)	(0.035)	(0.033)	(0.058)	(0.084)	(0.042)	(0.038)
Intercept	8.700***	9.805***	10.034***	10.531***	9.525***	10.690***	10.342***	10.633***	10.541***	10.168***
	(0.664)	(0.459)	(0.279)	(0.104)	(0.139)	(0.102)	(0.261)	(0.243)	(0.242)	(0.386)
Observations	485	526	779	3,195	2,206	1,742	1,001	592	410	517

*p<0.1; **p<0.05; ***p<0.01

Table S16: EJ Attribute: %Hispanic (Urban)

	EPA Region									
	(1)	(2)	(3)	(4)	(5)	(6)	(7)	(8)	(9)	(10)
Prop. Poverty	−1.182***	−0.681***	−0.447***	−0.607***	−0.955***	−0.513***	−0.440***	−1.471***	−0.357**	−1.119***
	(0.174)	(0.082)	(0.098)	(0.054)	(0.059)	(0.067)	(0.126)	(0.161)	(0.156)	(0.085)
Prop. White	1.142***	0.347***	0.419***	0.204***	0.377***	−0.041	0.165**	−1.321***	1.008***	−0.052
	(0.106)	(0.037)	(0.050)	(0.028)	(0.031)	(0.041)	(0.072)	(0.193)	(0.124)	(0.053)
Prop. Hispanic	0.517***	−0.032	−0.324***	0.301***	−0.708***	−0.268***	−0.802***	−1.557***	0.687***	−0.255***
	(0.146)	(0.053)	(0.090)	(0.047)	(0.049)	(0.045)	(0.135)	(0.223)	(0.172)	(0.051)
Pop. Density	−0.536***	−0.092***	−0.281***	−0.659***	−0.198***	−0.692***	−1.859***	−0.774***	−0.297***	−0.117***
	(0.055)	(0.010)	(0.040)	(0.038)	(0.027)	(0.053)	(0.142)	(0.121)	(0.062)	(0.025)
PM2.5 Z Score	−0.878***	−0.493***	−0.569***	−0.389***	−0.553***	−0.254***	−0.602***	−0.535***	−0.779***	−0.235***
	(0.056)	(0.027)	(0.026)	(0.016)	(0.025)	(0.018)	(0.049)	(0.049)	(0.033)	(0.009)
Intercept	7.639***	9.162***	9.202***	10.112***	9.828***	10.621***	10.310***	10.671***	9.060***	10.076***
	(0.186)	(0.430)	(0.166)	(0.089)	(0.127)	(0.108)	(0.228)	(0.266)	(0.361)	(0.410)
Observations	3,151	6,839	5,057	13,792	11,746	8,985	2,900	2,487	2,793	10,975

*p<0.1; **p<0.05; ***p<0.01

Table S17: EJ Attribute: %Poverty (Rural)

	EPA Region									
	(1)	(2)	(3)	(4)	(5)	(6)	(7)	(8)	(9)	(10)
Prop. Poverty	2.064***	1.140***	0.687***	0.311***	0.913***	0.585***	0.480**	1.092***	0.642**	0.549**
	(0.408)	(0.273)	(0.256)	(0.072)	(0.156)	(0.126)	(0.199)	(0.407)	(0.303)	(0.277)
Prop. non-White	−0.902*	−0.429***	−0.493**	0.044	−0.496***	−0.073	0.050	−0.058	0.155	−0.306**
	(0.536)	(0.159)	(0.195)	(0.041)	(0.106)	(0.063)	(0.174)	(0.172)	(0.156)	(0.138)
Pop. Density	−12.372**	−2.051*	−1.314	−5.051***	−1.529	−2.662**	−3.373	−1.566	−4.350	−7.586***
	(5.979)	(1.199)	(1.057)	(1.373)	(0.994)	(1.312)	(2.517)	(3.586)	(3.914)	(2.712)
PM2.5 Z Score	−0.251**	−0.122	−0.264***	−0.375***	−0.310***	−0.375***	−0.488***	−0.107	−0.301***	−0.104***
	(0.116)	(0.117)	(0.080)	(0.033)	(0.035)	(0.032)	(0.058)	(0.084)	(0.041)	(0.036)
Intercept	9.773***	10.051***	10.084***	10.423***	10.318***	10.631***	10.612***	10.698***	10.447***	10.780***
	(0.198)	(0.455)	(0.113)	(0.099)	(0.062)	(0.068)	(0.113)	(0.158)	(0.121)	(0.356)
Observations	485	526	779	3,195	2,206	1,742	1,001	592	410	517

*p<0.1; **p<0.05; ***p<0.01

Table S18: EJ Attribute: %Poverty (Urban)

	EPA Region									
	(1)	(2)	(3)	(4)	(5)	(6)	(7)	(8)	(9)	(10)
Prop. Poverty	−1.113***	−0.686***	−0.463***	−0.638***	−0.847***	−0.509***	−0.437***	−1.400***	−0.311**	−1.145***
	(0.173)	(0.082)	(0.098)	(0.054)	(0.059)	(0.067)	(0.127)	(0.163)	(0.156)	(0.085)
Prop. non-White	−0.879***	−0.356***	−0.477***	−0.137***	−0.524***	−0.098***	−0.337***	0.149	−0.676***	−0.146***
	(0.075)	(0.033)	(0.047)	(0.027)	(0.029)	(0.034)	(0.066)	(0.097)	(0.091)	(0.036)
Pop. Density	−0.522***	−0.093***	−0.313***	−0.636***	−0.245***	−0.706***	−1.977***	−0.737***	−0.322***	−0.121***
	(0.055)	(0.010)	(0.039)	(0.038)	(0.027)	(0.053)	(0.142)	(0.122)	(0.062)	(0.025)
PM2.5 Z Score	−0.889***	−0.496***	−0.579***	−0.383***	−0.594***	−0.256***	−0.607***	−0.527***	−0.776***	−0.238***
	(0.056)	(0.027)	(0.026)	(0.016)	(0.025)	(0.018)	(0.050)	(0.049)	(0.034)	(0.009)
Intercept	8.753***	9.506***	9.620***	10.320***	10.164***	10.588***	10.428***	9.401***	10.093***	10.036***
	(0.163)	(0.429)	(0.159)	(0.083)	(0.132)	(0.090)	(0.231)	(0.199)	(0.346)	(0.405)
Observations	3,151	6,839	5,057	13,792	11,746	8,985	2,900	2,487	2,793	10,975

*p<0.1; **p<0.05; ***p<0.01

Table S19: EJ Attribute: %White (Rural)

	EPA Region									
	(1)	(2)	(3)	(4)	(5)	(6)	(7)	(8)	(9)	(10)
Prop. Poverty	2.064***	1.140***	0.687***	0.311***	0.913***	0.585***	0.480**	1.092***	0.642**	0.549**
	(0.408)	(0.273)	(0.256)	(0.072)	(0.156)	(0.126)	(0.199)	(0.407)	(0.303)	(0.277)
Prop. White	0.902*	0.429***	0.493**	−0.044	0.496***	0.073	−0.050	0.058	−0.155	0.306**
	(0.536)	(0.159)	(0.195)	(0.041)	(0.106)	(0.063)	(0.174)	(0.172)	(0.156)	(0.138)
Pop. Density	−12.372**	−2.051*	−1.314	−5.051***	−1.529	−2.662**	−3.373	−1.566	−4.350	−7.586***
	(5.979)	(1.199)	(1.057)	(1.373)	(0.994)	(1.312)	(2.517)	(3.586)	(3.914)	(2.712)
PM2.5 Z Score	−0.251**	−0.122	−0.264***	−0.375***	−0.310***	−0.375***	−0.488***	−0.107	−0.301***	−0.104***
	(0.116)	(0.117)	(0.080)	(0.033)	(0.035)	(0.032)	(0.058)	(0.084)	(0.041)	(0.036)
Intercept	8.872***	9.623***	9.592***	10.467***	9.822***	10.558***	10.663***	10.640***	10.602***	10.474***
	(0.546)	(0.476)	(0.217)	(0.105)	(0.121)	(0.081)	(0.199)	(0.221)	(0.178)	(0.366)
Observations	485	526	779	3,195	2,206	1,742	1,001	592	410	517

*p<0.1; **p<0.05; ***p<0.01

Table S20: EJ Attribute: %White (Urban)

	EPA Region									
	(1)	(2)	(3)	(4)	(5)	(6)	(7)	(8)	(9)	(10)
Prop. Poverty	−1.113***	−0.686***	−0.463***	−0.638***	−0.847***	−0.509***	−0.437***	−1.400***	−0.311**	−1.145***
	(0.173)	(0.082)	(0.098)	(0.054)	(0.059)	(0.067)	(0.127)	(0.163)	(0.156)	(0.085)
Prop. non-White	0.879***	0.356***	0.477***	0.137***	0.524***	0.098***	0.337***	−0.149	0.676***	0.146***
	(0.075)	(0.033)	(0.047)	(0.027)	(0.029)	(0.034)	(0.066)	(0.097)	(0.091)	(0.036)
Pop. Density	−0.522***	−0.093***	−0.313***	−0.636***	−0.245***	−0.706***	−1.977***	−0.737***	−0.322***	−0.121***
	(0.055)	(0.010)	(0.039)	(0.038)	(0.027)	(0.053)	(0.142)	(0.122)	(0.062)	(0.025)
PM2.5 Z Score	−0.889***	−0.496***	−0.579***	−0.383***	−0.594***	−0.256***	−0.607***	−0.527***	−0.776***	−0.238***
	(0.056)	(0.027)	(0.026)	(0.016)	(0.025)	(0.018)	(0.050)	(0.049)	(0.034)	(0.009)
Intercept	7.874***	9.150***	9.143***	10.182***	9.640***	10.490***	10.092***	9.550***	9.418***	9.890***
	(0.176)	(0.429)	(0.164)	(0.086)	(0.135)	(0.092)	(0.238)	(0.211)	(0.353)	(0.405)
Observations	3,151	6,839	5,057	13,792	11,746	8,985	2,900	2,487	2,793	10,975

*p<0.1; **p<0.05; ***p<0.01

SI References

References

[1] Mariana C Arcaya, Gabriel Schwartz, and SV Subramanian. "A multilevel modeling approach to understanding residential segregation in the United States". en. In: *Environment and Planning B: Urban Analytics and City Science* 45.6 (Nov. 2018). Publisher: SAGE Publications Ltd STM, pp. 1090–1105. ISSN: 2399-8083. DOI: 10.1177/2399808318760858. URL: https://doi.org/10.1177/2399808318760858 (visited on 07/28/2024).

[2] Thomas J. Cooke. "Geographic context and concentrated urban poverty within the united states". In: *Urban Geography* 20.6 (Aug. 1999). Publisher: Routledge, pp. 552–566. ISSN: 0272-3638. DOI: 10.2747/0272-3638.20.6.552. URL: https://www.tandfonline.com/doi/abs/10.2747/0272-3638.20.6.552 (visited on 07/28/2024).

[3] Rachel E. Dwyer. "Contained Dispersal: The Deconcentration of Poverty in US Metropolitan Areas in the 1990s". en. In: *City & Community* 11.3 (Sept. 2012). Publisher: SAGE Publications, pp. 309–331. ISSN: 1535-6841. DOI: 10.1111/j.1540-6040.2012.01405.x. URL: https://doi.org/10.1111/j.1540-6040.2012.01405.x (visited on 07/28/2024).

[4] Thomas De Graaff, Raymond J.C.M. Florax, Peter Nijkamp, and Aura Reggiani. "A General Misspecification Test for Spatial Regression Models: Dependence, Heterogeneity, and Nonlinearity". en. In: *Journal of Regional Science* 41.2 (2001). _eprint: https://onlinelibrary.wiley.com/doi/pdf/10.1111/0022-4146.00216, pp. 255–276. ISSN: 1467-9787. DOI: 10.1111/0022-4146.00216. URL: https://onlinelibrary.wiley.com/doi/abs/10.1111/0022-4146.00216 (visited on 11/06/2023).

[5] Melanie M. Wall. "A close look at the spatial structure implied by the CAR and SAR models". In: *Journal of Statistical Planning and Inference* 121.2 (Apr. 2004), pp. 311–324. ISSN: 0378-3758. DOI: 10.1016/S0378-3758(03)00111-3. URL: https://www.sciencedirect.com/science/article/pii/S0378375803001113 (visited on 05/03/2024).

[6] Peter Congdon. *Applied Bayesian Modelling*. Newark, UNITED KINGDOM: John Wiley & Sons, Incorporated, 2014. ISBN: 978-1-118-89505-4. URL: http://ebookcentral.proquest.com/lib/nyulibrary-ebooks/detail.action?docID=1695071 (visited on 05/05/2024).

[7] Arthur Getis. "Spatial Autocorrelation". en. In: *Handbook of Applied Spatial Analysis: Software Tools, Methods and Applications*. Ed. by Manfred M. Fischer and Arthur Getis. Berlin, Heidelberg: Springer, 2010, pp. 255–278. ISBN: 978-3-642-03647-7. DOI: 10.1007/978-3-642-03647-7_14. URL: https://doi.org/10.1007/978-3-642-03647-7_14 (visited on 07/13/2023).

[8] Roger S. Bivand, Edzer Pebesma, and Virgilio Gómez-Rubio. "Modelling Areal Data". en. In: *Applied Spatial Data Analysis with R*. Ed. by Roger S. Bivand, Edzer Pebesma, and Virgilio Gómez-Rubio. Use R! New York, NY: Springer, 2013, pp. 263–318. ISBN: 978-1-4614-7618-4. DOI: 10.1007/

978-1-4614-7618-4_9. URL: https://doi.org/10.1007/978-1-4614-7618-4_9 (visited on 11/06/2023).

[9] Paula Moraga. *Chapter 7 Spatial neighborhood matrices — Spatial Statistics for Data Science: Theory and Practice with R*. en. URL: https://www.paulamoraga.com/book-spatial/spatial-neighborhood-matrices.html (visited on 08/30/2023).

[10] Håvard Rue, Sara Martino, and Nicolas Chopin. "Approximate Bayesian inference for latent Gaussian models by using integrated nested Laplace approximations". en. In: *Journal of the Royal Statistical Society: Series B (Statistical Methodology)* 71.2 (2009). _eprint: https://onlinelibrary.wiley.com/doi/pdf/10.1111/j.1467-9868.2008.00700.x, pp. 319–392. ISSN: 1467-9868. DOI: 10.1111/j.1467-9868.2008.00700.x. URL: https://onlinelibrary.wiley.com/doi/abs/10.1111/j.1467-9868.2008.00700.x (visited on 07/29/2024).

[11] Marta Blangiardo, Michela Cameletti, Gianluca Baio, and Håvard Rue. "Spatial and spatio-temporal models with R-INLA". In: *Spatial and Spatio-temporal Epidemiology* 4 (Mar. 2013), pp. 33–49. ISSN: 1877-5845. DOI: 10.1016/j.sste.2012.12.001. URL: https://www.sciencedirect.com/science/article/pii/S1877584512000846 (visited on 05/06/2024).

[12] Håvard Rue, Sara Martino, Finn Lindgren, Daniel Simpson, and A Riebler. *R-INLA: Approximate Bayesian Inference Using Nested Laplace Approximations*. Trondheim, Norway, 2013. URL: https://www.r-inla.org/ (visited on 07/29/2024).

[13] Diba Khana, Lauren M. Rossen, Holly Hedegaard, and Margaret Warner. "A BAYESIAN SPATIAL AND TEMPORAL MODELING APPROACH TO MAPPING GEOGRAPHIC VARIATION IN MORTALITY RATES FOR SUBNATIONAL AREAS WITH R-INLA". In: *Journal of data science : JDS* 16.1 (Jan. 2018), pp. 147–182. ISSN: 1680-743X. URL: https://www.ncbi.nlm.nih.gov/pmc/articles/PMC5839164/ (visited on 07/29/2024).

[14] Romi Satria, Jonathan Aguero-Valverde, and Maria Castro. "Spatial analysis of road crash frequency using Bayesian models with Integrated Nested Laplace Approximation (INLA)". In: *Journal of Transportation Safety & Security* 13.11 (Nov. 2021). Publisher: Taylor & Francis _eprint: https://doi.org/10.1080/19439962.2020. pp. 1240–1262. ISSN: 1943-9962. DOI: 10.1080/19439962.2020.1726542. URL: https://doi.org/10.1080/19439962.2020.1726542 (visited on 07/29/2024).

[15] Jiaqi Teng, Shuzhen Ding, Huiguo Zhang, Kai Wang, and Xijian Hu. "Bayesian spatiotemporal modelling analysis of hemorrhagic fever with renal syndrome outbreaks in China using R-INLA". en. In: *Zoonoses and Public Health* 70.1 (2023). _eprint: https://onlinelibrary.wiley.com/doi/pdf/10.1111/zph.12999, pp. 46–57. ISSN: 1863-2378. DOI: 10.1111/zph.12999. URL: https://onlinelibrary.wiley.com/doi/abs/10.1111/zph.12999 (visited on 07/29/2024).

[16] Loni Philip Tabb, Ana V. Diez Roux, Sharrelle Barber, Suzanne Judd, Gina Lovasi, Andrew Lawson, and Leslie A. McClure. "Spatially varying racial inequities in cardiovascular health and the contribution of individual- and neighborhood-level characteristics across the United States: The REasons for geographic and racial differences in stroke (REGARDS) study".

eng. In: *Spatial and Spatio-Temporal Epidemiology* 40 (Feb. 2022), p. 100473. ISSN: 1877-5853. DOI: 10.1016/j.sste.2021.100473.

[17] Julian Besag, Jeremy York, and Annie Mollié. "Bayesian image restoration, with two applications in spatial statistics". en. In: *Annals of the Institute of Statistical Mathematics* 43.1 (Mar. 1991), pp. 1–20. ISSN: 1572-9052. DOI: 10.1007/BF00116466. URL: https://doi.org/10.1007/BF00116466 (visited on 07/29/2024).

[18] Virgilio Gómez-Rubio. *Bayesian inference with INLA*. Boca Raton, FL: Chapman & Hall/CRC Press, 2020. ISBN: 978-1-138-03987-2. URL: http://becarioprecario.bitbucket.io/inla-gitbook/index.html (visited on 07/29/2024).

[19] Douglas Bates, Martin Maechler, Mikael Jagan, Timothy A. Davis, George Karypis, Jason Riedy, and Jens Oehlschlägel. *Matrix: Sparse and Dense Matrix Classes and Methods*. Apr. 2024. URL: https://cran.r-project.org/web/packages/Matrix/index.html (visited on 07/29/2024).

[20] R Core Team. *R: The R Project for Statistical Computing*. 2014. URL: https://www.r-project.org/ (visited on 07/29/2024).
